# Release of Intracellular Calcium Stores Facilitates Coxsackievirus Entry into Polarized Endothelial Cells

**DOI:** 10.1371/journal.ppat.1001135

**Published:** 2010-10-07

**Authors:** Rebecca A. Bozym, Stefanie A. Morosky, Kwang S. Kim, Sara Cherry, Carolyn B. Coyne

**Affiliations:** 1 Department of Microbiology and Molecular Genetics, University of Pittsburgh, Pittsburgh, Pennsylvania, United States of America; 2 Division of Pediatric Infectious Diseases, Johns Hopkins University School of Medicine, Baltimore, Maryland, United States of America; 3 Department of Microbiology, Penn Genome Frontiers Institute, University of Pennsylvania, Philadelphia, Pennsylvania, United States of America; Northwestern University, United States of America

## Abstract

Group B coxsackieviruses (CVB) are associated with viral-induced heart disease and are among the leading causes of aseptic meningitis worldwide. Here we show that CVB entry into polarized brain microvasculature and aortic endothelial cells triggers a depletion of intracellular calcium stores initiated through viral attachment to the apical attachment factor decay-accelerating factor. Calcium release was dependent upon a signaling cascade that required the activity of the Src family of tyrosine kinases, phospholipase C, and the inositol 1,4,5-trisphosphate receptor isoform 3. CVB-mediated calcium release was required for the activation of calpain-2, a calcium-dependent cysteine protease, which controlled the vesicular trafficking of internalized CVB particles. These data point to a specific role for calcium signaling in CVB entry into polarized endothelial monolayers and highlight the unique signaling mechanisms used by these viruses to cross endothelial barriers.

## Author Summary

Enteroviruses are associated with a number of diverse syndromes such as myocarditis, febrile illness, and are the main causative agents of aseptic meningitis. No effective therapeutics exist to combat non-poliovirus enterovirus infections. A better understanding of the mechanisms by which these viruses infect host cells could lead to the design of effective therapeutic interventions. In this study, we found that intracellular calcium stores in polarized endothelial monolayers are depleted upon exposure to coxsackievirus B (CVB) and that this release is mediated by viral attachment to its receptor decay-accelerating factor. We also discovered that the calcium release requires the activation of signaling molecules involved in calcium signaling such as Src tyrosine kinases, phospholipase C, and the inositol 1,4,5-trisphosphate receptor isoform 3 on the ER membrane. Furthermore, we found that a calcium-activated cystein protease, calpain-2, was activated and necessary for proper viral trafficking inside the cell. Interestingly, we found that this signaling cascade was critical for CVB internalization into the endothelium, but was not involved in CVB entry into the epithelium. This is an important advance in our understanding of how enteroviruses hijack host endothelial cell signaling mechanisms in order to facilitate their entry and eventual spread.

## Introduction

Coxsackievirus B (CVB), a member of the enterovirus family, is associated with a number of diverse syndromes including aseptic meningitis, myocarditis, febrile illness, and diabetes [1]. CVBs are transmitted via the fecal-oral route and encounter the polarized epithelium lining the gastrointestinal tract early in infection. Following dissemination, CVBs likely access secondary sites of infection via transmission through an endothelial monolayer such as that of the blood-brain barrier (BBB) and/or venous endothelium. Thus, although both polarized epithelial and endothelial cells function to prevent pathogen access to the interstitium, CVBs have developed strategies to subvert these barriers in order to promote their entry and/or dissemination. We have shown that CVB entry into polarized intestinal epithelial cells requires the activation of specific intracellular signaling molecules to promote viral endocytosis [2], [3]. However, it remains unclear if CVB also requires the initiation of host cell signaling to facilitate its entry (a process involving both endocytosis and vesicular trafficking) into the endothelium and whether the same signals are required between the epithelium and endothelium.

The binding of viruses to receptors on host cells often initiates elaborate signaling pathways aimed at facilitating viral uptake. The coxsackievirus and adenovirus receptor (CAR) mediates attachment by all six CVB serotypes [4], but is inaccessible to viruses on the luminal surface due to its localization within intercellular tight junctions [5]. For this reason, polarized cells are often resistant to infection by a number of CVB isolates [5]. Decay accelerating factor (DAF) is a glycosylphosphatidylinositol (GPI)-anchored membrane protein shown to bind several isolates of CVB (−1, −3, and −5) [4], [6], [7], [8], [9] and promote their infection of polarized cells [5]. As DAF is a GPI-anchored protein, it is localized to the apical surface of polarized cells and is accessible to virus in the lumen. In addition to providing a convenient site for virus attachment, the GPI anchor of DAF also facilitates its association with cholesterol-enriched lipid microdomains [10]. Lipid rafts are enriched in a number of signaling molecules including receptor tyrosine kinases, the Src family of nonreceptor tyrosine kinases, small G proteins, and adenylyl cyclases (ACs) [11].

Although DAF is anchored to the outer leaflet of the plasma membrane via a GPI anchor (and thus does not contain an intracellular domain), DAF and other GPI-anchored membrane proteins can be induced to form larger raft patches upon lateral crosslinking (most commonly with antibodies) [12]. We have shown previously that CVB-induced DAF clustering is essential for downstream signaling events required to facilitate virus entry into polarized intestinal epithelial cells [2]. Two tyrosine kinases (Abl and Fyn) are activated by DAF clustering and both are required for CVB entry into polarized epithelial cells [2]. Although clustering of GPI-anchored proteins is most commonly associated with the initiation of tyrosine kinase-based signaling cascades, the release of intracellular calcium (Ca_i_^2+^) following lateral crosslinking of these receptors has also been documented [13]. Antibody-mediated crosslinking of DAF has been linked to the release of Ca_i_^2+^ [14], [15] as a means to initiate monocyte activation [16].

Calcium is one of the most prominent second messengers in the cell. It is involved in many signaling cascades that have diverse outcomes depending on the spatiotemporal aspects of the calcium release. For this reason, intracellular calcium (Ca_i_^2+^) homeostasis is under tight regulation by the cell. The free cytoplasmic calcium concentration is maintained around 50–100 nM whereas intracellular stores such as the ER (endoplasmic reticulum) maintain much higher free concentrations (µM amounts). Intracellular calcium levels rise upon a stimulus (such as ligand-receptor interaction on the cell surface) and often converge on phospholipase C (PLC), an enzyme that mediates the hydrolysis of phosphatidylinositol-4,5-bisphophate (PIP_2_) into diacylglycerol (DAG) and inositol 1,4,5-trisphosphate (IP_3_). IP_3_ diffuses through the cytoplasm and binds to IP_3_ receptors (IP_3_R) localized on the ER membrane. Cytoplasmic calcium levels are brought back down to basal concentrations by multiple calcium channels such as the plasma membrane Ca^2+^-ATPase (PMCA), as well as the sarco-endoplasmic reticulum ATPase (SERCA) pump. As Ca_i_^2+^ levels regulate a variety of cellular processes, it is not surprising that many viral pathogens have evolved strategies to exploit Ca^2+^-mediated signaling events to promote mechanisms required to facilitate viral entry, replication, and/or spread [17].

Our previous studies have highlighted the intracellular signals that regulate CVB entry into polarized epithelial cells [2], [18]. In the present study, we have defined the role of Ca_i_^2+^ in facilitating CVB entry into human brain microvascular endothelia cells (HBMEC), an *in vitro* model of the blood-brain barrier. These studies have revealed that CVB-induced clustering of DAF induces an immediate depletion of Ca_i_^2+^ stores. CVB-induced Ca_i_^2+^ mobilization is regulated by several host cell factors including the Src family of tyrosine kinases, PLC, and is mediated specifically by the IP_3_R isoform 3. We also show that the calpain family of Ca^2+^-activated proteases plays a role in mediating the trafficking of CVB-containing vesicles within the cell. Interestingly, we also find that Ca_i_^2+^ release is involved in mediating CVB entry into primary human aortic endothelial cells, but is not required for CVB entry into polarized epithelial cells, suggesting that the intracellular signaling molecules hijacked by CVB to facilitate entry are distinct between the endothelium and epithelium.

## Results

### The mechanism of CVB entry is distinct between the endothelium and epithelium

Nonenveloped viruses gain entry into host cells by endocytic mechanisms that may include clathrin- or caveolar-mediated endocytosis, and macropinocytosis [19]. Some of these pathways are dependent upon the activity of dynamin, a GTPase required for vesicle fission. In previous studies, we found that CVB entry into polarized intestinal epithelial Caco-2 cells was independent of dynamin II [2] and occurred by a pathway that incorporates aspects of both caveolar-mediated endocytosis and macropinocytosis [3]. Because of the unique aspects of this pathway, we determined whether CVB entry into HBMEC occurrs via a similar mechanism. [Unless otherwise stated, all experiments were performed with CVB3-RD, a DAF-binding isolate of CVB].

**Figure 1:**
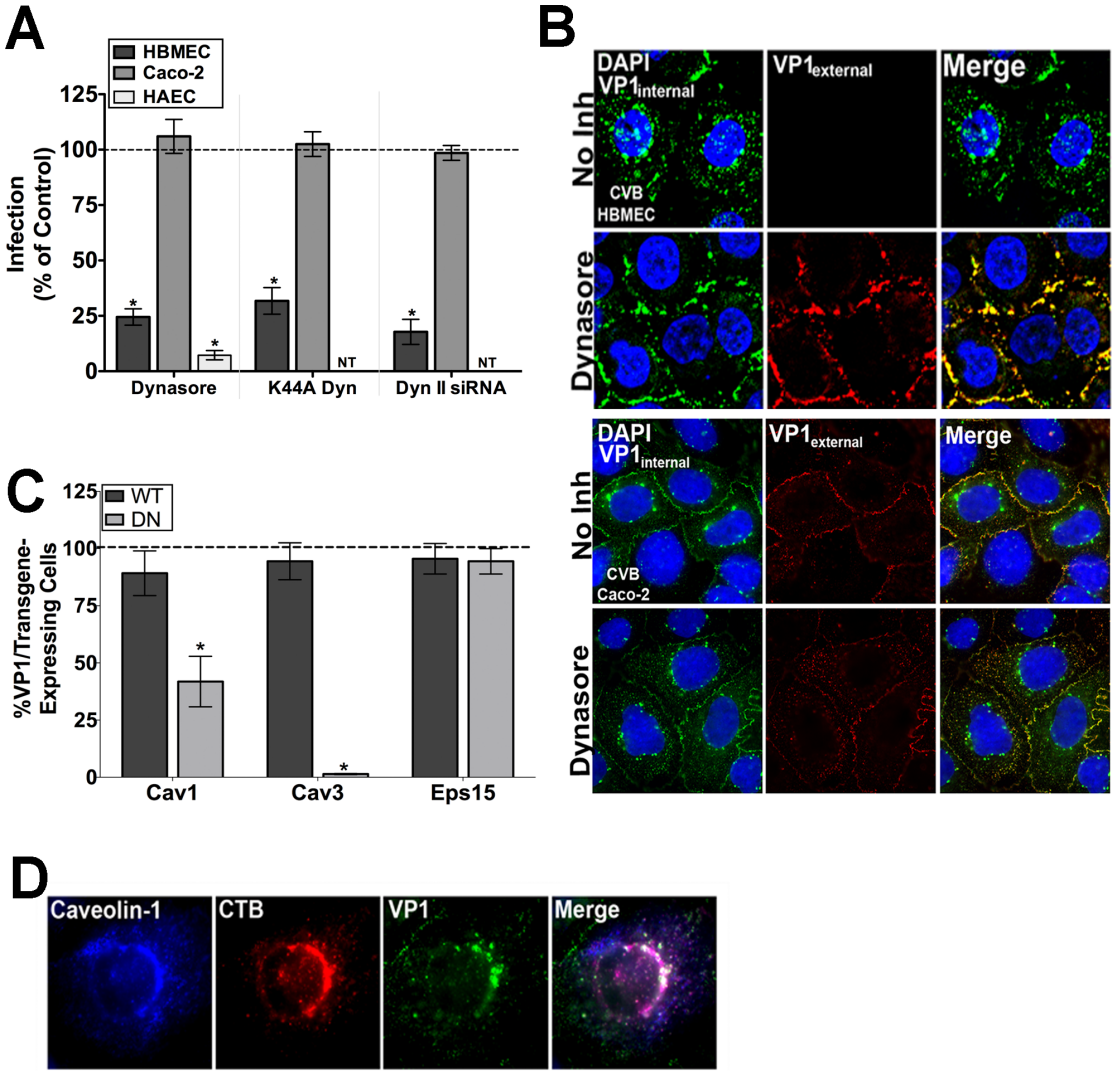
The CVB entry mechanism is distinct between polarized endothelia and epithelia. (**A**) Dynasore (100 µM), dynamin^K44A^, and dynamin II siRNA all significantly inhibit CVB infection in HBMEC and HAEC, but have no effect on CVB infection in Caco-2 cells. Data are normalized to DMSO control, wild-type dynamin II, or control siRNA-infected cells. (**B**) Immunofluorescence-based assay for viral internalization in HBMEC and Caco-2 cells pre-treated with DMSO (control) or dynasore and exposed to CVB (MOI = 50) for 1 hr at 37°C. Red fluorescence (or overlapping red and green fluorescence) indicates virus on the cell surface; green fluorescence in the absence of red indicates internalized virus. (**C**) HBMEC monolayers expressing dominant-negative or wild-type forms of caveolin-1, caveolin-3, or Eps15 were exposed to CVB (MOI = 1) and stained for VP1 at 14 hr p.i. The graph shows the number of transfected cells expressing VP1 as a percent of control infections (dashed line). (**D**) HBMEC monolayers exposed to CVB (MOI = 50) and Alexa-Fluor cholera toxin-B (CTB – red) were stained for caveolin-1 (blue) and VP1 (green) at 60 min p.i.

First, we used three independent methods to alter dynamin II activity –*(1)* dynasore, a cell-permeable inhibitor of dynamin [20], (2) a dominant-negative mutant of dynamin II (dynamin II K44A) [21], and (3) siRNA-mediated depletion of dynamin II (Supplemental Figure S5)–and determined the effects of this alteration on CVB infection of HBMEC and Caco-2 cells. Under all of these conditions, CVB infection of Caco-2 cells was unaffected (Figure 1A) while all methods significantly reduced infection of HBMEC by CVB (Figure 1A). Moreover, using a fluorescence-based assay for viral internalization that discriminates between virus on the cell surface and that which has internalized [18], we confirmed that dynasore specifically inhibited CVB entry into HBMEC (Figure 1B, top) while having no effect on its entry into Caco-2 cells (Figure 1B, bottom). Interestingly, CVB infection of primary human aortic endothelial cells (HAEC) was also inhibited by dynasore treatment (Figure 1A), suggesting that the route of entry of CVB into the aortic endothelium may be similar to that in the CNS microvasculature.

We next determined the effect of dominant-negative mutants of various endocytic pathways for their effects on CVB infection of HBMEC. These studies revealed that CVB infection of HBMEC was significantly impaired when mutants of the caveolar pathway were expressed (caveolin-1 and -3), consistent with what we observed previously in Caco-2 cells [2] (Figure 1C). Furthermore, immunofluorescence microscopy revealed colocalization of cytoplasmic CVB-containing vesicles with caveolin-1 and cholera toxin B (a marker of the caveolar pathway) (Figure 1D). In contrast, infection was unaffected by expression of a mutant of the clathrin endocytic pathway (Eps15) in HBMEC (Figure 1C). These data indicate that the mechanism of CVB entry into the endothelium is clathrin-independent, and likely occurs via a dynamin- and caveolar-dependent pathway. In contrast, entry into the epithelium occurs via a clathrin-and dynamin-independent, but caveolin-dependent pathway [2]. Taken together, these findings point to a divergent mechanism of endocytosis between the endothelium and epithelium.

### Ca^2+^ is required for CVB infection of HBMEC

We have shown that CVB entry into polarized epithelial Caco-2 cells requires the activation of intracellular signaling molecules to facilitate viral endocytosis [2] and are initiated by viral attachment to DAF on the apical cell surface. Because our current findings indicate that CVB entry occurs via disparate mechanisms between HBMEC and Caco-2 cells (Figure 1A, 1B), we investigated the host cell signaling molecules involved in facilitating CVB entry into HBMEC and whether these molecules were unique between these cell types.

**Figure 2:**
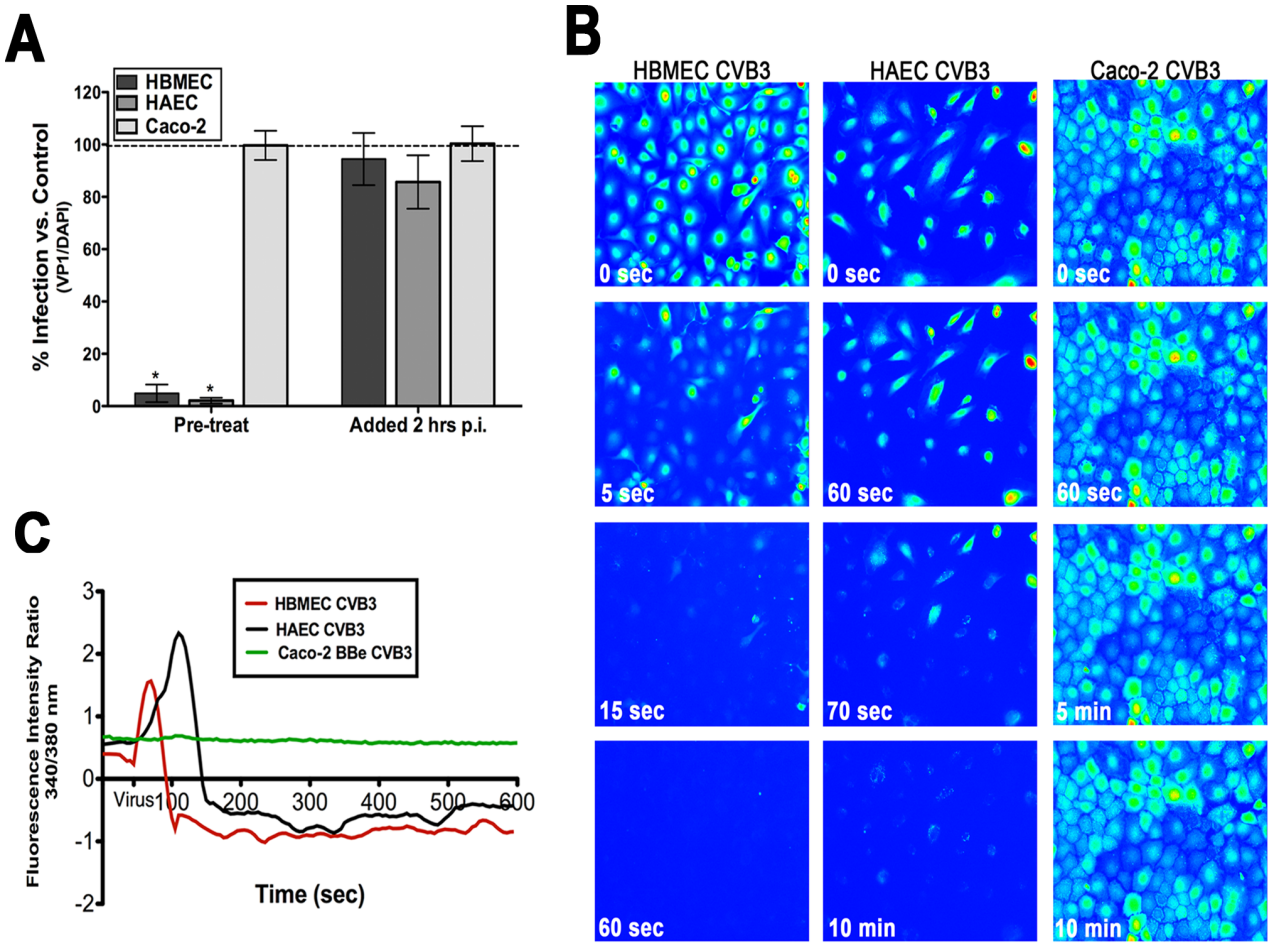
Intracellular Ca^2+^ is required and mobilized in endothelia but not epithelia. (**A**) HBMEC, primary HAEC, or Caco-2 cells were treated with Bapta-AM [in low calcium (<300 µM Ca^2+^)-containing media] and infected with CVB (5 PFU/cell) for 14 hrs. Inhibitor was added to cultures 1 hour prior to infection (pre-treat) or 2 hours p.i. (post-treat). The graph indicates the percentage of cells expressing VP1 compared to no inhibitor controls (dashed line). (**B**) HBMEC (left), HAEC (middle), and Caco-2 (right) monolayers were loaded with Fura-2 AM and fluorescent images (excitation 340 and 380 nm) were taken every 5 seconds prior to and following the addition of CVB at MOI = 50 (at t = 55 seconds). Shown are images (pseudocolored) captured at the indicated times. (**C**) Fluorescence intensity ratio (340/380 nm) of Fura-2-AM versus time of HBMEC (red), HAEC (black), and Caco-2 cells (green) exposed to CVB3.

As DAF signaling has been associated with the release of Ca_i_^2+^ [14], [15], we determined whether CVB infection of HBMEC was sensitive to manipulation of Ca_i_^2+^ stores. We found that in cells pretreated with Bapta-AM (a chelator of intracellular calcium), infection was significantly reduced compared to no inhibitor controls (Figure 2A). Interestingly, Bapta-AM lost its inhibitory effect when added at a post-entry time point [2 hrs post infection (p.i.), Figure 2A], indicating that Ca^2+^ may be required for events occurring at or very close to the time of virus entry. Similar results were obtained in primary human aortic endothelial cells (HAEC) (Figure 2A). We found that this effect was specific for CVB as Bapta-AM had no effect on vesicular stomatitis virus (VSV) infection of HBMEC (Supplemental Figure S1A). In addition, Bapta-AM had no effect on CVB infection of intestinal epithelial Caco-2 cells either pre- or post-treatment (Figure 2A), indicating that the role of Ca^2+^ in early events associated with CVB is specific to polarized endothelia.

### CVB entry induces the rapid release of Ca_i_^2+^ in HBMEC

Because we observed that Ca^2+^ chelation inhibited CVB infection (Figure 2A) of HBMEC, we next determined the kinetics of CVB-mediated Ca_i_^2+^ release in real-time. To do this, we used live-cell imaging of HBMEC loaded with the ratiometric Ca_i_^2+^ indicator Fura-2 AM. This allowed for the tracking of individual cells to pinpoint the precise timeframe during which intracellular Ca^2+^ store depletion occurred. Images were captured every 5 sec at both excitation wavelengths for Fura (340/380 nm, emission 510). Following a brief period to establish baseline levels of Ca_i_^2+^ (t = 50 sec), CVB (MOI = 50) was added directly to monolayers. To prevent Ca^2+^ influx due to alterations in membrane permeability, monolayers were bathed in Ca^2+^-free HEPES-buffered saline. The addition of CVB resulted in an almost immediate release (<20sec) of Ca_i_^2+^ [shown in still images spanning 1 min from virus addition (Figure 2B, Supplemental Movie S1)]. As quickly as 15 sec following the addition of CVB, the majority of cells have been almost completely depleted of Ca_i_^2+^ [shown in the graphical representation (Figure 2C)]. Of particular significance, this depletion occurred at a time point prior to viral uncoating (Supplemental Figure S1D) and the production of viral proteins (Supplemental Figure S1B, S1C), which have previously been shown to induce Ca_i_^2+^ release at late stages of virus replication [22].

We next tested whether primary human aortic endothelial cells (HAEC) were also depleted of Ca_i_^2+^ in response to CVB exposure. In some cases, microvasculature and arterial endothelial cells differ in the degree of tight junction function and in their responsiveness to calcium ionophores [23]. Furthermore, myocarditis and dilated cardiomyopathy are often associated with CVB infection and CVB may infect aortic endothelial cells during cardiac infections [24], [25]. Interestingly, we observed the depletion of Ca_i_^2+^ in response to CVB exposure of HAEC similar to that observed in HBMEC (Figure 2B, C). However, CVB-induced depletion of Ca_i_^2+^ proceeded at a more gradual pace (<120 sec) in HAEC compared to HBMEC (Supplemental Movie S2).

Consistent with our findings that Bapta-AM had no effect on CVB infection Caco-2 cells (Figure 1A), we found that CVB entry had no effect on Ca_i_^2+^ levels in these cells (Figure 2B, 2C, Supplemental Movie S3). These data indicate that the role of Ca_i_^2+^ in mediating CVB entry is specific to the endothelium and suggest that there may be unique signaling molecules activated between the endothelium and epithelium.

### DAF mediates CVB-induced Ca_i_^2+^ release

**Figure 3:**
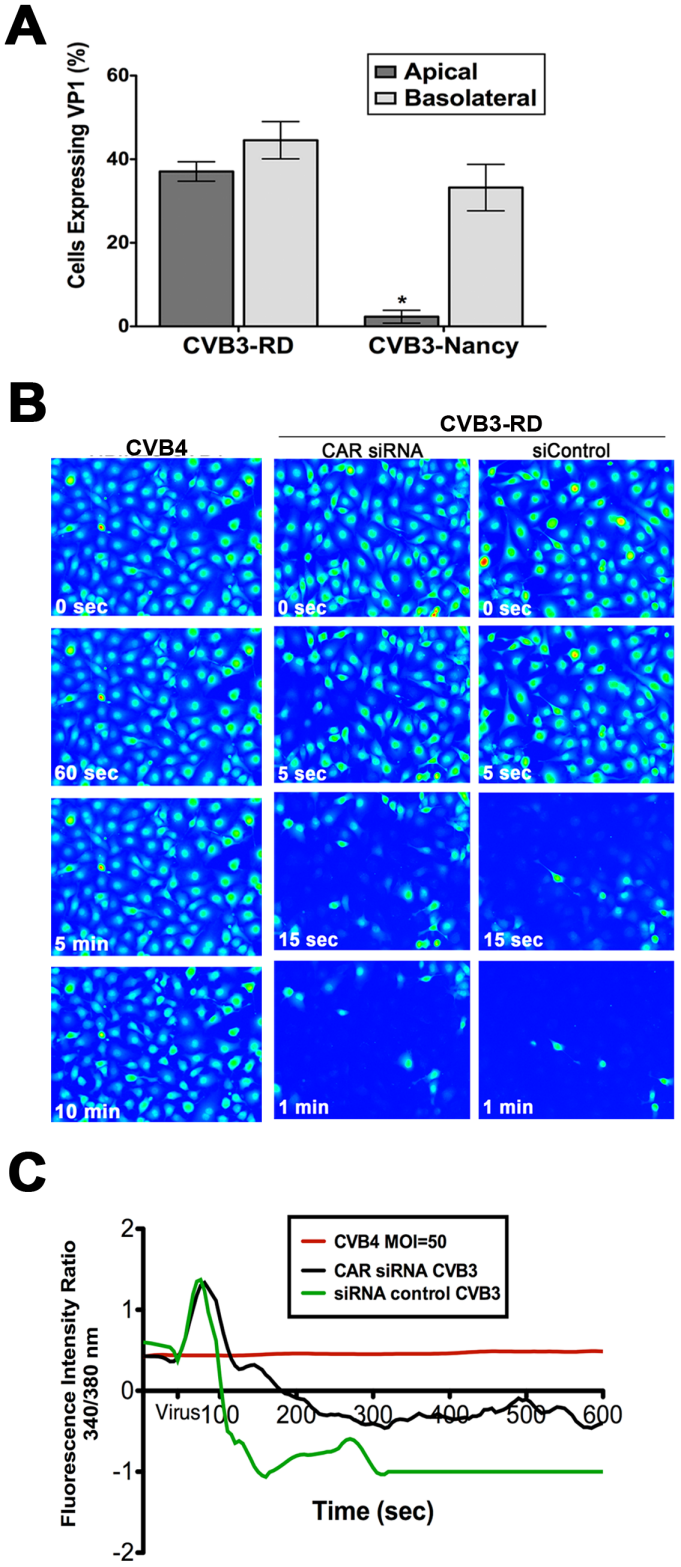
DAF mediates CVB-induced Ca_i_^2+^ release. (**A**) HBMEC grown in transwells were exposed to CVB3-RD or CVB3-Nancy (a non-DAF binding isolate) on the apical and basolateral side, infected for 14 hrs, and fixed and stained for the VP1. Shown are the percentage of infected cells (normalized to DAPI-stained nuclei). (**B**) HBMEC monolayers loaded with Fura-2 AM were exposed to CVB4, a non-DAF binding CVB isolate, at t = 55 seconds with MOI = 100 (left). HBMEC monolayers transfected with CAR (middle) or control (right) siRNAs were loaded with Fura-2AM and exposed to CVB3-RD (MOI = 100, t = 55 seconds). Shown are images (pseudocolored) captured at the indicated times. (**C**) Fluorescence intensity ratio of (340/380 nm) of Fura-2AM versus time of HBMEC monolayers exposed to CVB4 (red) or control- (green) or CAR-transfected (black) siRNAs exposed to CVB3.

Although DAF is known to mediate CVB attachment to and infection of polarized epithelial cells [5], little is known regarding its role in mediating infection of the polarized endothelium. Consistent with what has been observed in polarized intestinal monolayers [5], we found that a non-DAF binding CVB isolate (CVB-Nancy) was incapable of infecting HBMEC from the apical surface (Figure 3A) and DAF siRNA (Supplemental Figure S5) inhibited binding and infection by CVB (Supplemental Figure S2A). This would indicate that DAF plays an essential role in facilitating CVB infection of the endothelium [likely because CAR is also sequestered in the tight junctions of HBMEC (Supplemental Figure S2B) and is not exposed to virus approaching from the apical domain].

To determine whether CVB-DAF interactions are involved in Ca_i_^2+^ store depletion in HBMEC, we used a non-DAF binding isolate of CVB (CVB4) and determined its effects on Ca_i_^2+^ release. We found that CVB4 did not induce any noticeable Ca_i_^2+^ release (Figure 3B,C) as CVB4-exposed cells retained their Ca_i_^2+^levels throughout the entire 10 min time course (Supplemental Movie S4).

To exclude any CAR-dependent signaling events upstream of CVB-induced Ca_i_^2+^ release, we determined the extent of Ca_i_^2+^ release in HBMEC transfected with CAR siRNA and exposed to DAF-binding CVB. We found that CAR siRNA (which led to a >90% depletion of CAR expression, Supplemental Figure S5) had no effect on CVB-induced Ca_i_^2+^ release in HBMEC (Figure 3B, 3C, and Movie S5). These data support a role for DAF, but not CAR, in the induction of Ca_i_^2+^ release in response to CVB entry.

### IP_3_R type 3 and PLCγ are required for calcium mobilization in response to CVB

**Figure 4:**
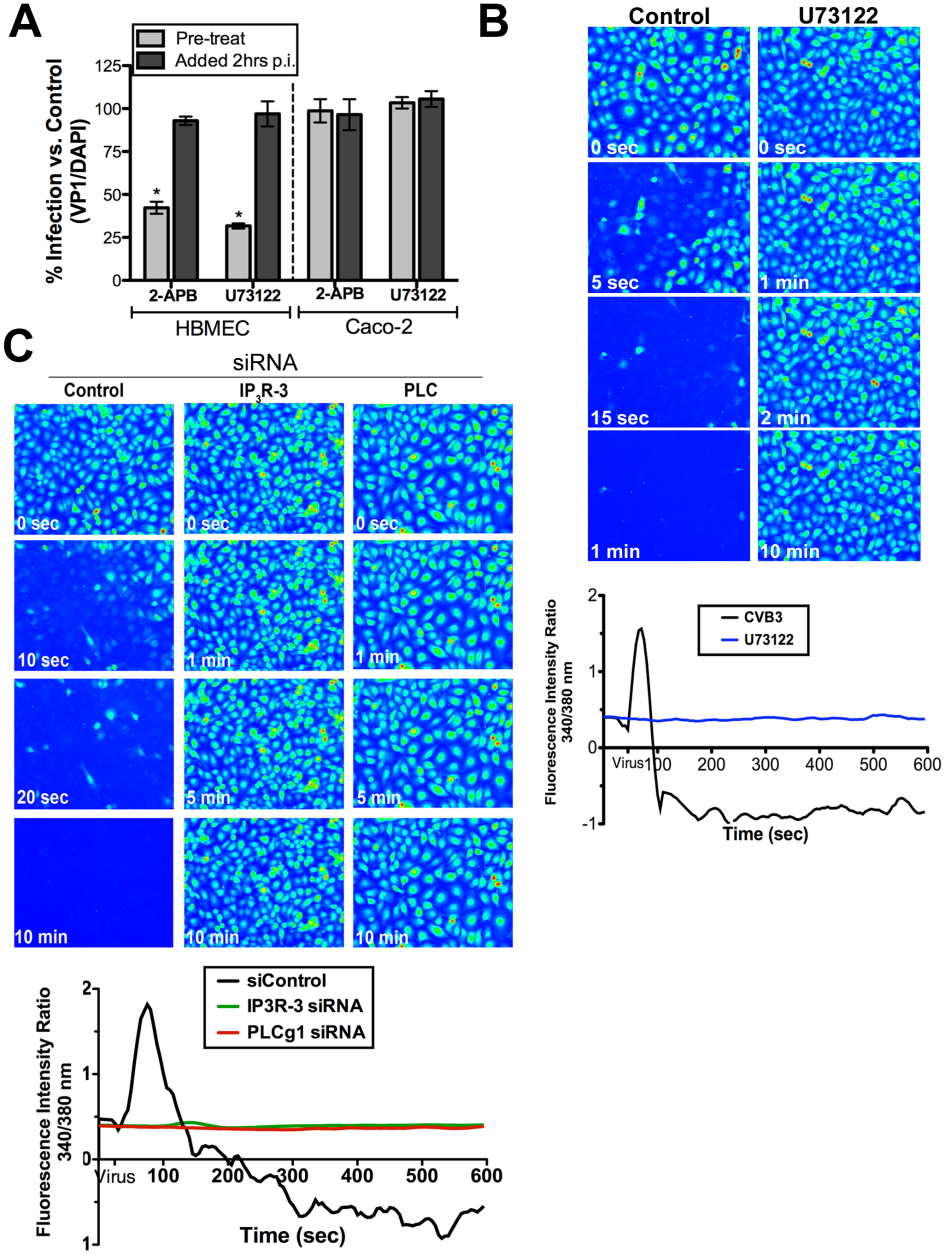
PLCγ and IP_3_R-3 are involved in CVB-induced depletion of Ca_i_^2+^ stores. (**A**) HBMEC (left) or Caco-2 (right) cells treated with 2-APB or U73122 1 hour prior to infection (pre-treat) or 2 hours p.i. (post-treat) were infected with CVB (MOI = 1) for 14 hrs (HBMEC) or 7 hrs (Caco-2). Graph represents percentage of total cells expressing VP1 normalized to no-inhibitor controls (dashed line). (**B**) **Top:** Still images of HBMEC pre-treated with U73122, loaded with Fura-2 AM, and exposed to CVB (MOI = 50, t = 55 seconds). Images were pseudo colored for Ca_i_^2+^ visualization. **Bottom:** Fluorescence intensity ratio (340/380 nm) of Fura-2AM versus time in HBMEC exposed to CVB in the absence (black) or presence of U73122 (blue). (**C**) **Top:** Still images captured at the indicated times in HBMEC transfected with control, IP_3_R-3, or PLCγ-1 siRNAs, loaded with Fura-2AM and exposed to CVB (MOI = 50, t = 55 seconds). **Bottom:** Intensity ratio plot (340/380 nm) of HBMEC loaded with Fura-2AM and transfected with control, IP_3_R-3, or PLCγ -1 siRNA and exposed to CVB (MOI = 50, t = 55 seconds).

Ca_i_^2+^ mobilization is often initiated by ligand interaction with cell surface receptors which can lead to the activation of intracellular signaling molecules such as tyrosine kinases, and/or PLCs (reviewed in [26]). These molecules can either act directly to increase IP_3_ levels (i.e. PLCs) or increase IP_3_R sensitivity to IP_3_ binding in the absence of the generation of new IP_3_ (i.e. tyrosine kinases) [27], [28], [29]. To determine whether CVB-induced Ca_i_^2+^ release required the activation of PLC (and the subsequent IP_3_R-mediated release of Ca_i_^2+^), we tested the effects of 2-APB (an inhibitor of IP_3_R channels) and U73122 (a specific PLC inhibitor) for their effects on CVB infection in HBMEC. We found that pre-treatment of cells with both 2-APB and U73122 led to a significant reduction in CVB infection (Figure 4A). In contrast, exposure of cells to both inhibitors at a post-entry time point (2 hrs p.i.) had no effect. We also found that U73122 inhibited Ca_i_^2+^ release in response to CVB entry (Figure 4B). Consistent with our findings that CVB entry into Caco-2 does not require Ca_i_^2+^ (Figure 1B), we found that 2-APB and U73122 had no effect on CVB infection in Caco-2 cells at either pre- or post-entry time points (Figure 4A).

Although we observed an inhibition of Ca_i_^2+^release in cells treated with U73122, this inhibitor targets a wide range of PLC isoforms. For this reason, we determined whether PLCγ1 (PLCG1), a known mediator of Ca_i_^2+^ release, was specifically involved in CVB-induced Ca_i_^2+^ release using siRNA-mediated knockdown. We found that depletion of PLCγ1 significantly inhibited CVB-mediated release of Ca_i_^2+^ (Figure 4, Supplemental Figure S5, Movie S6).

The majority of Ca_i_^2+^ oscillations within cells occur via bursts, sparks, or waves produced by the activation of IP_3_R. Three IP_3_R have been identified in mammalian cells that differ in their affinity for IP_3_, but whose specific functions remain uncertain (reviewed in[26]). The expression pattern of the different IP_3_R subtypes between tissues is likely responsible for the variety of patterns associated with Ca_i_^2+^ release between cell types (and may ultimately determine the physiological outcomes of this release). Endothelial cells generally express all three IP_3_R isoforms to some degree [30]–[31]. We employed the use of siRNAs to specifically knockdown IP_3_R isoforms expressed in HBMEC– IP_3_R-1, IP_3_R-2, and IP_3_R-3 (Supplemental Figure S5). Whereas knockdown of IP_3_R-1 and IP_3_R-2 had modest effects on CVB-induced Ca_i_^2+^ release (Supplemental Figure S3 and Movie S7 and S8), knockdown of IP_3_R-3 resulted in a complete inhibition of Ca_i_^2+^ release upon exposure to CVB (Figure 4C, supplemental Movie S9). These data indicate that while IP_3_R-1 and IP_3_R-2 may play minor roles in mediating CVB-induced Ca_i_^2+^ release, IP_3_R-3 is likely the critical IP_3_R isoform involved.

### Src family kinases are upstream of CVB-induced Ca_i_^2+^ release

**Figure 5:**
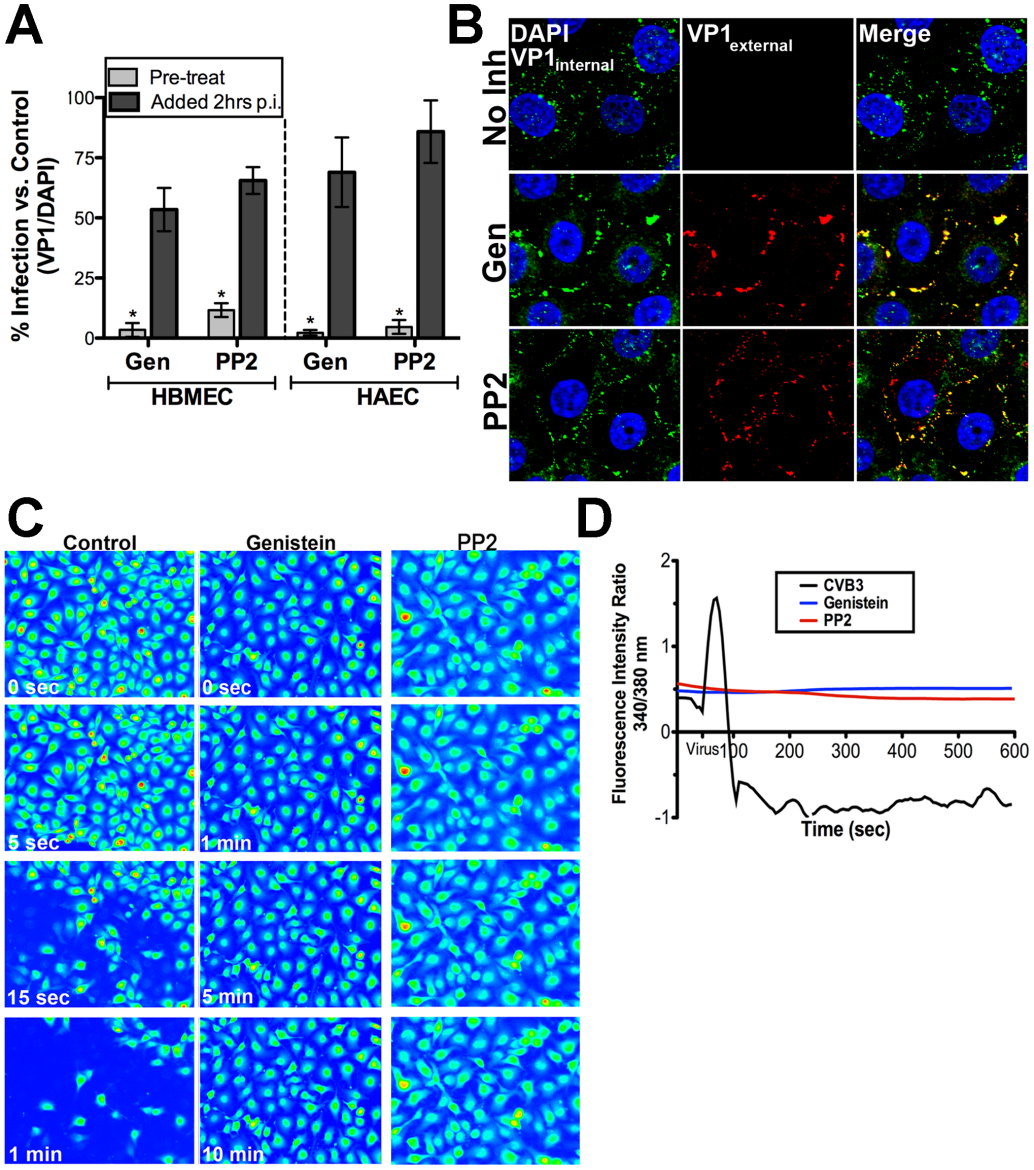
Src Family Tyrosine kinases are upstream of intracellular Ca^2+^ release in response to CVB3. (**A**) HBMEC (left) or HAEC (right) monolayers were pre-treated with genistein or PP2 1 hr before (pre-treat) exposure to CVB3 or 2 hrs p.i. and infected for 14 hrs (HBMEC) or 7 hrs (Caco-2). Shown are the percentage of infected cells (normalized to DAPI-stained nuclei) normalized to no inhibitor controls. (**B**) Immunofluorescence-based assay for viral internalization in HBMEC pre-treated with no inhibitor, genistein, or PP2 and exposed to CVB3 (MOI = 100) for 60 min and stained as decsribed in Materials and Methods. Blue = DAPI-stained nuclei, red = externalized virus (VP1external), and green = internalized virus (VP1internal). (**C**) Still images captured at the indicated times in HBMEC monolayers treated with control, genistein, or PP2, loaded with Fura-2AM, and exposed to CVB. (**D**) Intensity ratio plot (340/380 nm) of control (no inhibitor)-, genistein-, or PP2-treated HBMEC loaded with Fura-2AM and exposed to CVB3 (t = 55 seconds).

We have shown that CVB exploits DAF-mediated tyrosine signaling pathways to surmount the epithelial barrier in order to gain entry into polarized epithelial cells [2]. Because we observed that CVB-induced Ca_i_^2+^ release in HBMEC required DAF-binding (Figure 3B), we tested whether tyrosine kinases might play a role upstream of Ca_i_^2+^ release in HBMEC. We found that tyrosine kinase activity was required for CVB infection of HBMEC as treatment of cells with the non-specific tyrosine kinase inhibitor genistein reduced both CVB infection (Figure 5A) and entry (Figure 5B). Because genistein targets a broad range of tyrosine kinases, we determined the effects of PP2 (a specific Src tyrosine kinase inhibitor) on CVB entry and infection. We found that PP2 significantly reduced CVB infection (Figure 5A) and entry (Figure 5A), indicating that Src family kinase activity is required for CVB entry into HBMEC (similar to our previous findings in Caco-2 cells).

Because tyrosine kinases, including members of the Src kinase family [28], [32], have been shown to function upstream of Ca_i_^2+^ release, we next determined whether tyrosine kinases and/or Src kinase activity was required to facilitate CVB-mediated Ca_i_^2+^ release. To do this, we pre-treated HBMEC with either genistein or PP2 and measured CVB-induced Ca_i_^2+^ release in real-time. We found that there was a profound inhibition of CVB-induced Ca_i_^2+^ release by both genistein and PP2 compared to controls (Figure 5C and D). We also found that genistein inhibited CVB-induced Ca_i_^2+^ release in HAEC, indicating a similar mechanism of release may exist between the microvasculature and arterial endothelium (Supplemental Figure S4). These data point to a role for Src family tyrosine kinase signaling in CVB-induced Ca_i_^2+^ release.

### Calpain activity is required for CVB trafficking

**Figure 6:**
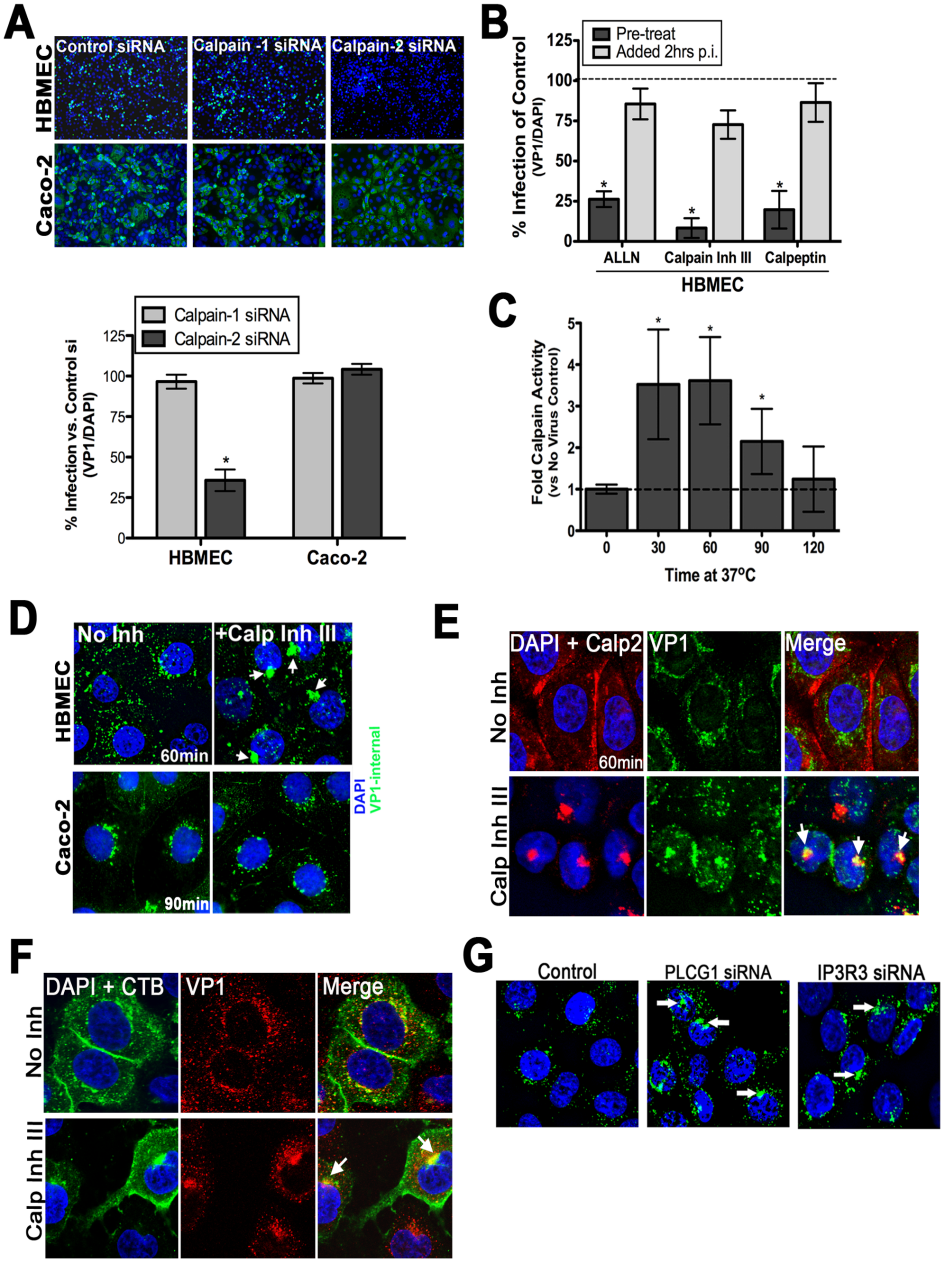
Calpain-2 is required for vesicular trafficking of internalized CVB. (**A**) **Top:** Representative images of HBMEC and Caco-2 monolayers transfected with control, calpain-1, or calpain-2 siRNAs and infected with CVB (MOI = 1) for 14 hrs (HBMEC) or 7 hrs (Caco-2). VP1 in green and DAPI-stained nuclei in blue. **Bottom:** Effect of calpain-1 or calpain-2 siRNA transfection on CVB infection of HBMEC (left) or Caco-2 (right) cells. Shown are the percentage of infected cells (normalized to DAPI-stained nuclei) normalized control siRNA-transfected cells (**B**) HBMEC monolayers were treated with the indicated calpain inhibitors and infected with CVB (MOI = 1) for 14 hrs. Inhibitor was added to cultures 1 hr before infection (pre-treat) or 2 hrs p.i. Dashed line indicates the infection level of control cells. (**C**) Calpain activity was measured in HBMEC infected with CVB (50 PFU/cell) for the indicated times. Dashed line indicates calpain activity in control (no virus) cells. (**D**) Immunofluorescence microscopy in HBMEC (top) and Caco-2 (bottom) exposed to CVB (MOI = 50) for 60 min and treated with DMSO (no inhibitor) or calpain inhibitor III. Green staining represents internalized virus. White arrows denote enlarged virus-contained vesicles in calpain inhibitor III-treated cells. (**E**) Immunofluorescence microscopy in HBMEC exposed to CVB (MOI = 50) for 60 min and treated with either control (No Inh) or with calpain inhibitor III. VP1 (green), calpain-2 (red), and DAPI (blue). White arrows denote enlarged virus-containing vesicles in calpain inhibitor III-treated cells that colocalize with calpain-2. (**F**) Immunofluorescence microscopy in HBMEC exposed to CVB (MOI = 50) and Alexa Fluor-488 conjugated cholera toxin B (CTB) for 60 min and treated with either control (No Inh) or with calpain inhibitor III. CTB (green), VP1 (red), and DAPI (blue). White arrows denote enlarged CTB and virus-containing vesicles in calpain inhibitor III-treated cells that colocalize with calpain-2. (**G**) Immunofluorescence microscopy in HBMEC transfected with control, PLCγ-1, or IP_3_R-3 siRNAs and stained for internalized CVB (MOI = 50, in green) and DAPI (in blue) at 60 minutes p.i. White arrows denote enlarge virus-containing vesicles in PLCγ-1 and IP_3_R-3 siRNA treated cells.

We recently performed an RNAi screen for host factors involved in CVB infection of HBMEC and identified calpain-2, a Ca_i_^2+^-dependent cysteine protease, as being required for CVB infection of HBMEC (CB Coyne and S Cherry, unpublished data). Members of the calpain family are activated by release of Ca_i_^2+^ and can be categorized into two subfamilies–µ-calpains (eg., calpain-1) are activated by micromolar concentrations of Ca_i_^2+^; and m-calpains (eg., calpain-2) are activated by millimolar concentrations of Ca_i_^2+^ [reviewed in [33]]. We found that whereas siRNA-mediated knockdown of calpain-2 decreased CVB infection significantly, downregulation of calpain-1 had little effect (Figure 6A, bottom, and Supplemental Figure S5). In accordance with our findings that Ca_i_^2+^ plays no role in CVB entry into Caco-2 cells (Figure 2A, 2B, 4A), we found that reduction of calpain-2 expression had no effect on CVB infection of Caco-2 cells (Figure 6A, bottom).

To confirm the role of calpain-2 in mediating CVB infection of HBMEC, we treated cells with three known inhibitors of calpains–ALLN, calpeptin, and calpain inhibitor III—and found that they significantly reduced infection by CVB in HBMEC (Figure 6B). Likewise, HAEC pre-treated with calpain inhibitor III also had a significant reduction in infection (Supplemental Figure 6B). In contrast, calpain activity was not required for CVB infection in Caco-2 cells (Supplemental Figure S6A). Although inhibition of calpain activity exhibited potent reduction in CVB infection when cells were pretreated with inhibitor, we found that this effect did not occur when calpain inhibitors were added at post-entry time points (2 hr p.i.) (Figure 6B and Supplemental Figure S6B). These findings suggest that calpain activity is required early in the life cycle of CVB (possibly at or near the time of viral entry). Consistent with this, we found that calpains were activated by 30 min p.i., (Figure 6C), likely coincident with CVB entry and following the release of Ca_i_^2+^ induced by CVB binding.

To further define the mechanism by which calpain-2 facilitates CVB infection we used a fluorescence-based assay for viral internalization. Using this assay, we found that while calpain activity was not required for viral endocytosis into the cytoplasm, it was required for proper vesicular trafficking as we observed the appearance of large CVB-containing intracellular vesicles >500 nm in diameter (much larger than the average size of endosomes) when calpain activity was inhibited in HBMEC (Figure 6D and Supplemental Figure S6C,D). These large structures remained in the cytoplasm for extended periods of time (>5 hours, not shown) whereas in untreated cells these vesicles traveled to a perinuclear compartment by 60–120 min (where the release of viral RNA likely occurs). In contrast, inhibition of calpain activity had no effect on CVB entry or trafficking within Caco-2 cells (Figure 6D). We found that these long-lived cytoplasmic virus-containing vesicles were heavily associated with calpain-2 (Figure 6E) and cholera toxin B (Figure 6F). However, we did not observe any significant colocalization between internalized CVB particles and calpain-2 in control cells (Figure 6E). Although calpain-2 has been shown to regulate endosomal trafficking [34], [35], it remains unclear if calpain associates with endosomal membranes for any significant length of time. Consistent with a potential transient interaction between calpain-2 and endosomal membrane protein components, we also did not observe any significant colocalization between calpain-2 and a component of early endosomes (Rab5 GTPase) (Supplemental Figure S6F). Taken together, these data suggest that Ca_i_^2+^ release results in the specific activation of calpain-2 that in turn facilitates the trafficking of virus-containing vesicles within the cytoplasm to a perinuclear location for uncoating and RNA replication to ensue. Furthermore, the role of calpain-2 is specific to the endothelium as inhibition of calpain activity had no effect on CVB infection of intestinal epithelial cells.

Because both PLCγ1 and IP_3_R-3 appeared to play significant roles in mediating Ca_i_^2+^ signaling in response to CVB entry, we next determined whether they were also involved in facilitating CVB entry and/or trafficking. Similar to our findings when calpain activity was inhibited, we found that knockdown of PLCγ1 and IP_3_R-3 also altered the ability of internalized CVB particles to properly traffic within the cytoplasm and led to the accumulation of long-lived CVB-containing vesicles within the cytoplasm (Figure 6G and Supplemental Figure 6E). These data suggest that the PLCγ1- and IP_3_R-3-dependent Ca_i_^2+^ release induced by CVB entry is required for the activation of calpain-2 to facilitate vesicular trafficking of internalized viral particles.

## Discussion

Many viral pathogens have developed strategies to subvert the barriers presented by epithelia and endothelia in order to infect the host or spread to secondary sites of infection. The CNS and heart are common sites of CVB secondary infection. In order to infect these tissues, circulating CVB would require passage through or infection of the endothelium in order to traffic from the circulatory system into the underlying tissue (through a process that likely requires apical DAF engagement). Our previous studies have established that CVB enters polarized cells by endocytic mechanisms that require activation of specific intracellular signaling molecules including the tyrosine kinases Fyn and Abl [2], [18]. Here we show how CVB specifically exploits Ca_i_^2+^-mediated signaling events in order to facilitate its entry into polarized endothelial cells. We provide evidence that CVB-induced Ca_i_^2+^ release is triggered by virus binding to DAF and involves the activity of the Src family of tyrosine kinases, PLCγ1, and the expression of a specific IP_3_R isoform, IP_3_R-3. The release of Ca_i_^2+^ induced by CVB is required for the subsequent activation of calpain-2, which facilitates CVB vesicular trafficking. We also show that the Ca_i_^2+^-dependence of CVB entry is specific to the endothelium and is not involved in mediating CVB entry into the epithelium. The necessity for Ca_i_^2+^ release in endothelia, but not epithelia, demonstrates that the entry of CVB (and likely other viral pathogens) is mediated by cell-type-specific intracellular signals that may differ between polarized cell types.

Viral receptors often facilitate host cell signaling events required for virus entry. Our results show that CVB-induced Ca_i_^2+^ release is triggered by CVB-DAF interactions and occurs even in the absence of CAR expression (Figure 3B). This is not surprising given that DAF is located within lipid raft domains [2] and is in close proximity to signaling molecules such as receptor tyrosine kinases and PLCs [11]. CD59, another GPI-anchored receptor, leads to the recruitment of tyrosine kinases, the heterotrimeric G protein Gα_i_2, and PLCγ1 upon antibody-induced lateral crosslinking. This crosslinking leads to the activation of PLCγ1 and a subsequent burst in Ca_i_^2+^ [13]. It is therefore likely that CVB exploits Ca_i_^2+^-associated signaling events associated with DAF crosslinking in order to facilitate its entry and intracellular trafficking.

Several viruses have been shown to manipulate host cell Ca_i_^2+^ homeostasis in order to promote their entry and/or replication [22], [27], [36], [37], [38], [39], [40], [41]. Herpes simplex virus (HSV) has been shown to utilize a transient increase in intracellular Ca_i_^2+^ concentration triggered by receptor binding to promote its internalization [42]. Similar to our findings with CVB, HSV-induced Ca_i_^2+^ release is mediated by the activation of PLC and subsequent activation of IP_3_R [43]. In addition, depletion of ER-derived Ca^2+^ stores inhibits infection of SV40, suggesting that there may be modulation of Ca_i_^2+^ homeostasis induced during its entry [44]. It is thus becoming clear that viruses from several unrelated families have developed strategies to target Ca_i_^2+^ signaling in order to facilitate their entry.

Tyrosine kinase signaling often functions upstream of Ca_i_^2+^ release to activate PLCγ and/or directly phosphorylate IP_3_Rs. The role for Src family tyrosine kinases in the release of Ca_i_^2+^ is clear–mice deficient in Fyn kinase are devoid of certain types of Ca_i_^2+^ release [45], c-Src-specific antibodies inhibit PLCγ-dependent Ca_i_^2+^ release [46], [47], and Fyn directly phosphorylates IP_3_R to permit extended Ca_i_^2+^ release upon IP_3_ binding [32] [28]. We found that Src kinases were critical for CVB-induced Ca_i_^2+^ release. Interestingly, our previous work has shown that Src kinases, specifically Fyn, mediate the entry of CVB into intestinal Caco-2 cells [2]. However, here we show that Ca_i_^2+^ plays no role in CVB entry into Caco-2 cells, indicating that although Src kinases facilitate CVB entry into both polarized epithelia and endothelia, they target divergent downstream targets to do so. We also show that inhibition of Src kinase activity prevents CVB entry into HBMEC (Figure 5B). However, the point in the entry process that was inhibited by Src kinase inhibition (e.g. cell surface) was unique from what we observed by inhibiting calpains or PLCγ and IP_3_R-3 expression (e.g. intracellular viral trafficking). As Src kinases function in many aspects of endocytosis [48]–[49], these data indicate that they likely serve multiple functions in regulating CVB entry into HBMEC beyond that of Ca_i_^2+^ release. Taken together, our findings indicate that Src kinases are pivotal regulators of CVB-induced signal propagation in the endothelium and epithelium, but likely target unique downstream effector molecules to facilitate CVB entry.

Src kinases have been shown to directly phosphorylate IP_3_Rs in order to modulate their affinity for IP_3_s and/or alter their gating kinetics [32] [28]. There are three isoforms of the IP_3_R in mammalian cells, but the precise function and cellular requirement for each isoform remains uncertain. Although functional redundancy likely exists between isoforms, IP_3_R-specific localization, gating, and regulation by ligands/proteins for specific cell processes contributes to isoform-specific signaling. Our results indicate that Ca_i_^2+^ release downstream of CVB-induced DAF clustering is mediated via activation of IP_3_R-3, as siRNA targeting IP_3_R-3 inhibited this release (Figure 4C). However, other Ca^2+^ channels may be involved as we cannot exclude the possibility that channels (such as store-operated cation channels or Ca^2+^-release activated channels) are activated via IP_3_R-3-mediated Ca^2+^ release to induce Ca^2+^ influx. Interestingly, caveolin-1 has been shown to directly bind IP_3_R-3 to regulate agonist-induced Ca_i_^2+^ release [50] and the endothelium of mice deficient in caveolin-1 display alterations in Ca_i_^2+^ fluxes (despite equivalent levels of IP_3_ production) [51]. As we found that CVB gains entry into HBMEC via a caveolar-dependent mechanism (Figure 1), it is conceivable that the activation of caveolar-mediated endocytosis induced by CVB entry alters the association between caveolin-1 and IP_3_R-3 to alter its gating properties and/or sensitivity to IP_3_ as a mechanism to promote Ca_i_^2+^ release.

We observed pronounced activation of calpain coincident with CVB entry (Figure 6) and calpain activity was required to regulate the trafficking of CVB-containing vesicles within the cell cytoplasm. Calpains are Ca^2+^-dependent cysteine proteases, most of which are ubiquitously expressed, and function in many cellular processes, although the vast majority of these functions are still largely unclear (reviewed in [33]). Calpain substrates can include cytoskeletal proteins, kinases and phosphatases, membrane-associated proteins including ion channels, and various transcription factors [33]. Several studies have linked calpains as important regulators of viral replication. Latently infected HIV-1 cells utilize Ca^2+^-dependent calpain activation in order to initiate viral replication [52], hepatitis C virus utilizes calpain activity in the cleavage of viral nonstructural proteins [53], and echovirus 1 requires calpains for an as-yet-unidentified facet of its replication [54]. In contrast to these other viruses, we find that calpain-2 is required at the time of CVB entry and has little role in post-entry events in the virus life cycle. The precise role for calpain-2 in regulating the trafficking of CVB-containing vesicles is uncertain. However, calpains have been implicated in endocytosis, particularly in the regulation of intracellular membrane fusion, and are associated with coated vesicles within the cytoplasm [34], [35], [55]. A role for calpain-2 in regulating vesicular fusion during CVB entry is supported by our observation that internalized CVB particles accumulate within enlarged cytoplasmic vesicles when calpain activity is inhibited. Additionally, calpains have also been associated with the remodeling of the actin cytoskeleton by targeting a variety of actin-associated components. Thus, calpains may facilitate CVB trafficking by modulating the actin cytoskeletal network for proper vesicular trafficking. Calpain-2 is activated by high levels of Ca_i_^2+^ (mM), consistent with the pronounced release of Ca_i_^2+^ induced during CVB entry. Moreover, we also observed the appearance of enlarged CVB-positive cytoplasmic vesicles when the expression of PLCγ1 and IP_3_R-3 were depleted, supporting a role for PLCγ1- and IP_3_R3-dependent Ca_i_^2+^ release upstream of calpain-2 activation.

Although many viral pathogens target polarized cells, little is known regarding the mechanisms used by viruses to enter polarized monolayers or whether these mechanisms might differ between the epithelium and endothelium. CVB entry into polarized epithelial cells is a complex process that involves the activation of a variety of intracellular signaling molecules that regulate distinct aspects of the viral internalization process [2], [3]. The results presented here show that CVB entry into polarized endothelial cells is regulated by a divergent intracellular signaling pathway than that in the epithelium–the mobilization of Ca_i_^2+^. Thus, CVB has evolved to hijack two distinct pathways in the endothelium and epithelium to bypass polarized cell barriers. These results provide an illustration of the complexities likely to be associated with viral internalization into polarized cells and may serve as a model for how other viral pathogens circumvent the barriers presented by polarized cell monolayers.

## Materials and Methods

### Cell culture and viruses

HBMEC were cultured in RPMI 1640 (Hyclone, Logan, Utah) with 10% FBS (Gibco, Grand Island, New York), 10% NuSerum (BD Biosciences, Bedford, MA), 100 U/ml of NEAA (nonessential amino acids), MEM vitamins, and sodium pyruvate (all Hyclone), 10 U/ml of PenStrep (Gibco), and 30 µg/ml of Endothelial Cell Growth Supplement (BD Biosciences) and have been described previously [56]. Primary HAEC were obtained from Lonza-Clonetics (Allendale, NJ) and cultured in EGM-2 media per manufacturer's instructions. Caco-2 (BBE clone) were purchased from the ATCC and grown in DMEM-H supplemented with 10% FBS and 10 U/ml PenStrep. CVB3-RD and CVB4 were expanded by infecting HeLa cells, purified through centrifugation in a sucrose gradient, and tittered by plaque assays on HeLa cells as described previously [2].

### Antibodies

Mouse anti-enterovirus VP1 (Ncl-Entero) was obtained from Novocastra Laboratories (New Castle upon Tyne, UK). Goat polyclonal antibodies to calpain-2 (N-19) was purchased from Santa Cruz Biotechnology (Santa Cruz, CA). Alexa fluor-conjugated secondary antibodies and cholera toxin B were purchased from Invitrogen (Carlsbad, CA).

### Inhibitors

Genistein (20 µM), ALLN (50 µM), calpeptin (10 µM), dideoxyadnesoine (5 µM), PP2 (10 µM), and calpain inhibitor III (5 µM) were purchased from Calbiochem (Gibbstown, NJ); U73122 (700 µM), Bapta-AM (10 µM), 2-APB (30 µM), and dynasore (100 µM) were purchased from Sigma (St. Louis, MO). Toxicity panels were performed to ensure inhibitors did not cause unwanted effects (Supplemental Figure S7).

### Immunofluorescence microscopy

HBMEC monolayers grown in collagen-coated chamber slides (BD Biosciences, San Jose, CA) were exposed to CVB in binding buffer for 1 hour at 16°C then washed and placed at 37°C to initiate entry (for entry experiments), or 14 hours at 37°C (for infection experiments). For entry experiments the cells were washed and fixed with 4% paraformaldehyde (PFA) and then incubated with primary VP1 antibody for 1 hour. Each well was then washed and incubated with the appropriate Alexa Fluor-594-conjugated antibody for 30 min. After another washing the cells were fixed again with 4% PFA, washed, permeabilized with 0.1% Triton-X 100 in PBS, incubated again with VP1 primary antibody for 1 hour at room temperature, washed, and subsequently incubated with Alexa Fluor-488-conjugated secondary antibody for 30 min, washed, and then mounted with Vectashield (Vector Laboratories, Burlingame CA). For infection experiments cells were exposed to virus at MOIs stated then washed and fixed with ice-cold methanol acetone (3∶1). Monolayers were then incubated with primary VP1 antibody for 1 hour, washed, and incubated with secondary Alexa Fluor–488-conjugated antibody. Cells were imaged on an Olympus IX81 inverted microscope equipped with a motorized stage for obtaining Z stacks. For virus entry experiments, images were captured with an Olympus PlanApo 60x/1.42 NA oil objective with z stacks (0.25 µM slices) and deconvolution performed by using the nearest neighbor function in Slidebook 5.0. Infection images were captured with an Olympus UplanApo 10x/0.4 NA objective and quantified using ImageJ (http://rsb.info.nih.gov/ij/) as a ratio of VP1^+^/DAPI^+^.

### Immunofluorescence-based assay for internalized virus

Immunoflorescence imaging for internalized viral particles was performed as described in detail previously [18]. Briefly, monolayers were exposed to CVB (50 particles/cell) and at the indicated times fixed in 4% PFA, washed in PBS containing 50 mM NH_4_Cl for 5 min, and incubated with monoclonal anti-VP1 antibody (NCL-ENTERO) for 1 h at RT. Cells were then washed and incubated with Alexa Fluor (AF) 594-conjugated secondary antibody. Following washing, cells were fixed again in 4% PFA, incubated for 5 min in PBS containing 50 mM NH_4_Cl, and permeabilized with 0.1% Triton X-100 for 10 min. Permeabilized monolayers were re-incubated with anti-VP1 antibody, washed, and incubated with AF 488-conjugated secondary antibody. Cells were mounted with Vectashield containing DAPI and images captured as described above.

### Ratiometric calcium imaging

Cells grown on collagen-coated glass bottom 35 mm dishes (MatTek Corp., Ashland, MA) were loaded with Fura-2 AM (1 µM - Invitrogen) for 30 min at 37°C. These culture conditions promoted the formation of polarized monolayers characterized by the asymmetric localization of apical and basolateral protein components (Supplemental Figure S2B). Cells were rinsed 3 times with Ca^2+^- and Mg^2+^-free PBS, bathed in a final volume of 1 ml. Images were captured on an Olympus IX81 motorized inverted microscope equipped with a Hamamatsu Orca-R2 CCD camera, Sutter Lambda 10-3 High Speed filter wheel system, and an Olympus UApo/340 20x objective with an N.A. of 0.75. Images were acquired using Slidebook 5.0 advanced imaging software. Selected cells were chosen (40 regions of interest (ROI)/dish) and images captured at both excitation 340 nm and 380 nm every 5 seconds for 10 minutes (experiments were performed a minimum of three times). Virus was added to dishes once baseline was established (t = 55 sec) at the specified MOIs. Intensity ratios for selected ROIs were calculated using Slidebook 5.0, and replicates averaged and plotted as a function of time. Images were pseudocolored (using Slidebook 5.0) in order to better visualize Ca_i_^2+^ mobilization with blue  =  low Ca_i_^2+^ and red  =  high Ca_i_^2+^.

### siRNA transfections

siRNAs were purchased from Dharmacon. HBMEC were transfected using HiPerFect (Qiagen, Valencia, CA) as described previously [3]. Reverse transfections were performed as follows– OptiMEM:HiPerfect complexes were incubated for 10 min with the indicated siRNAs and then added to cells in suspension (harvested following trypsinization) and incubated for 48–72 hours. In some cases, siRNAs were delivered by nucleofection [Nucleofector System (Amaxa) using Nucleofector solution V and program T023].

### RT-PCR

Total RNA was isolated with TRI Reagent Solution (Applied Biosystems, Foster City, CA) according to the manufacturer's protocol. For complementary DNA synthesis, 1 µg total RNA was used in a 20-µL reaction containing 1 mM deoxynucleotide triphosphates (dNTPs), 2.5 mM oligo dT or random hexamers (for CVB amplification), 1000 U/ml RNase inhibitor, 0.1 volume 10X buffer (supplied by manufacturer), and 2500 U/ml murine leukemia virus reverse transcriptase (Invitrogen, Carlsbad, CA). The reverse transcription (RT) reaction was carried out at 1 cycle in a thermal cycler at 42°C for 50 min, followed by 15 min incubation at 70°C. PCR for IP_3_R-2 was carried out with primers to the gene of interest (primer sequences can be found in Supplemental Figure S5B) and *Taq* DNA polymerase for 25 cycles. PCR products were separated on a 1% agarose gel containing ethidium bromide. Primer sequences are as follows: IP_3_R-2 (sense 5′-CTTGAAGATCTGGGGGATCA-3′ and antisense 5′-GTGCCTTCTTTTGCCTCTTG-3′); IP_3_R-1 (sense 5′-CAAGCGAGTTCCTGTTCTCC-3′ and antisense 5′-GTGGACTCCAGCTTCTCCTG-3′); GAPDH (sense 5′-ACCACCAACTGCTTAGCA-3′ and antisense 5′-CCCTGTTGCTGTAGCCAA-3′). CVB PCR was performed using a Maxim Biotech amplification kit for enteroviruses as per the manufacturer's instructions.

### Calpain activation assay

Calpain activity was assessed in HBMEC exposed to CVB (100 PFU/cell) at the indicated times using a fluorogenic calpain activity assay (Calbiochem). Briefly, control or CVB-exposed cells (at the indicated times) were lysed in RIPA buffer (without protease inhibitors) and incubated with fluorogenic calpain substrate for 15 min at room temperature. Fluorescence intensity measurements were acquired using a fluorescence plate reader (BioTek Synergy 4, BioTek) at an excitation wavelength of ∼360–380 nm and an emission wavelength of ∼440–460 nm. Readings were normalized to background (RIPA alone) controls and data presented as the fold change in calpain activity in CVB-exposed cells compared to no virus controls.

### Accession numbers

ID numbers for proteins/genes mentioned in the text (numbers were taken from GenBank at Pubmed): inositol 1,4,5-trisphosphate receptor 1 (ITPR1) 3708; inositol 1,4,5-trisphosphate receptor 3 (ITPR3) 3710; phospholipase C gamma-1 (PLCG1) 5335; decay accelerating factor (DAF or CD55) 1604; coxsackievirus and adenovirus receptor (CXADR) 1525; calpain-2 (CAPN2) 824; calpain-1 (CAPN1) 823; Tec kinase (TEC) 7006; dynamin (DNM1) 1759; dynamin II (DNM2) 1785; caveolin-1 (CAV1) 857; caveolin-3 (CAV3) 859; EPS15 2060.

## Supporting Information

Figure S1 CVB-induced Ca_i_^2+^ depletion occurs prior to uncoating and replication. **(A)** HBMEC were treated with Bapta-AM and infected with VSV (MOI = 1) for 8 hrs. Inhibitor was added to cultures 1 hr before (pre-treat) or 2 hrs after (post-treat). The graph indicates the percentage of cells expressing VSV-G compared to control (dashed line). **(B)** RT-PCR or **(C)** Western blot analysis of RNA/protein collected from HBMEC infected with CVB (10 PFU/cell) for the indicated times. Negative [(-) no infection] and positive [(+) overnight infection with CVB] are shown. **(D)** ^35^S-labeled virus particle at various stages of internalization were recovered by cell lysis with sucrose gradient lysis buffer (10mM Tris-HCl, pH 7.6, 1mM NaCl, 1mM EDTA, 1% NP40, 0.5% sodium dodecyl sulfate (SDS). Cell lysates were overlaid on linear 15-30% sucrose gradients and centrifuged at 39,000 rpm for 150 min at 4°C in a Beckman SW41Ti rotor. Fractions (400 µl) were collected from the top of the gradient and radioactivity was measured.
(0.32 MB TIF)

Figure S2 CAR is sequestered in the tight junctions of HBMEC. **(A)** HBMEC were transfected with control, CAR, or DAF siRNAs and exposed to S^35^-labeled CVB (12,000 cpms) at 16°C for one hour. Following binding, cells were washed, lysed, and radioactivity was counted. **(B)** Confocal micrographs of HBMEC immunostained for the basolateral-localized Na+/K+ ATPase pump (green) and the tight junction marker ZO-1 (red) (DAPI - blue). **(C)** Confocal micrographs of CAR (green) and ZO-1 (red, left), or E-cadherin (red, right).
(1.97 MB TIF)

Figure S3 IP_3_R-1 and -2 siRNAs have modest effects on CVB-induced Ca_i_^2+^ mobilization. **(A)** Intensity ratio graph of HBMEC transfected with control, IP_3_R-1, or IP_3_R-2 siRNAs, loaded with Fura-2AM and exposed to CVB (55 sec). **(B)** Still images of Fura-2-loaded HBMEC transfected with control, IP_3_R-1, or IP_3_R-2 siRNAs and exposed to CVB.
(3.27 MB TIF)

Figure S4 Tyrosine kinases are required for CVB-induced Ca_i_^2+^ release in HAEC. **(A)** Intensity ratio graph of HAEC pre-treated with control (no inhibitor) or genistein and exposed to CVB (55 sec). **(B)** Still images of Fura-2 loaded HAEC with or without genistein and exposed to CVB.
(1.54 MB TIF)

Figure S5 Efficacy of siRNA silencing in HBMEC. Western blot or RT-PCR analysis in HBMEC transfected with the indicated siRNAs: control (CON), calpain-1 (CALP1), calpain-2 (CALP2), CAR, DAF, dynamin II (DNMII), IP_3_R1, IP_3_R2, IP_3_R3, or PLCγ1 (PLCG1). For immunoblots, membranes were stripped and reprobed with GAPDH as a loading control. For RT-PCR, cDNA was amplified using GAPDH primers.
(0.39 MB TIF)

Figure S6 Calpain-2 is required for viral trafficking in HAEC and HBMEC. **(A)** Caco-2 monolayers were treated with the indicated calpain inhibitors and infected with CVB (MOI = 1) for 7hrs (Caco-2). Inhibitor was added to cultures 1 hr before infection (pre-treat) or 2 hrs p.i. Dashed line indicates the infection level of control cells. **(B)** Primary HAEC cells were treated with calpain inhibitor III and infected with CVB. Inhibitor was added to cultures 1 hr before infection (pre-treat) or 2 hrs p.i. **(C)** Quantification of vesicles (with diameter >500nM) in HBMEC in the absence or presence of calpain inhibitors. Data are presented as the percent of total cells containing vesicles >500nM in diameter (total number of cells counted - 75 for no inhibitor and 117 for calpain inhibitors). **(D)** Representative images of the quantification shown in (C). VP1 (green) and DAPI (blue). **(E)** Quantification of vesicles (with diameter >500nM) in HBMEC transfected with control siRNA, PLCG1 siRNA, and IP_3_R-3 siRNA. **(F)** Confocal images of HBMEC stained for calpain 2 (red) and stained with mouse monoclonal Rab5 GTPase (green) (2143, Cell Signaling Technology).
(1.63 MB TIF)

Figure S7 Toxicity panels for pharmacological inhibitors and siRNAs. **(A)** Extent of PI uptake in HBMEC following 7 hr incubation with the indicated inhibitors. Toxicity was calculated as the percent of cells positive for PI/total cells. **(B)** Induction of type I interferon signaling in HBMEC transfected with a luciferase reporter plasmid and then select siRNAs. Data are presented as a fold increase in comparison to control (no siRNA) levels.
(0.93 MB TIF)

Movie S1 Intracellular calcium store depletion is observed immediately after exposure of HBMEC monolayers to CVB3. Time-lapse movie of HBMEC loaded with Fura-2AM and exposed to CVB3 (50 PFU/ml) in real time. Movie is pseudocolored for better visualization of calcium (blue = low, red = high).
(6.36 MB MOV)

Movie S2 Intracellular calcium store depletion is also observed immediately after exposure of HAEC to CVB3. Time-lapse movie of HAEC monolayers loaded with Fura-2AM and exposed to CVB (50 PFU/cell) in real time. Movie is pseudocolored for better visualization of calcium (blue = low, red = high).
(8.60 MB MOV)

Movie S3 Epithelial Caco-2 monolayers exposed to CVB3 do not mobilize intracellular calcium. Time-lapse movie of Caco-2 monolayers loaded with Fura-2AM and exposed to CVB (50 PFU/cell) in real time. Movie was pseudocolored for better visualization of calcium (blue = low, red = high).
(10.37 MB MOV)

Movie S4 CVB4 does not induce intracellular calcium release of HBMEC monolayers. Time-lapse movie of HBMEC monolayers loaded with Fura-2AM and exposed to CVB4 (50 PFU/cell) in real time. Movie was pseudocolored for better visualization of calcium (blue = low, red = high).
(9.93 MB MOV)

Movie S5 Intracellular calcium store depletion in response to CVB3 is not dependent on CAR. Time-lapse movie of HBMEC monolayers transfected with CAR siRNA, loaded with Fura-2AM, and exposed to CVB3 (50 PFU/ml) after 1 min. Movie is pseudocolored for better visualization of calcium (blue = low, red = high).
(6.78 MB MOV)

Movie S6 PLCG1 is required for calcium store depletion in response to CVB3. Time-lapse movie of HBMEC monolayers transfected with PLCG1 siRNA, loaded with Fura-2AM, and then exposed to CVB3 (50 PFU/ml) after 1 min. Movie was pseudocolored for better calcium visualization (blue = low, red = high).
(10.32 MB MOV)

Movie S7 IP_3_R1 siRNA has a modest effect on calcium mobilization in response to CVB3. Time-lapse movie of HBMEC monolayers transfected with IP_3_R-1 siRNA, loaded with Fura-2AM, and exposed to CVB3 (MOI = 50) after 1 min. Movie was pseudocolored for better calcium visualization (blue = low, red = high).
(10.19 MB MOV)

Movie S8 IP_3_R-2 siRNA has a modest affect on calcium store depletion in response to CVB3. Time-lapse movie of HBMEC monolayers transfected with IP_3_R-2 siRNA, loaded with Fura-2AM, and exposed to CVB3 (MOI = 50) after 1 min. Movie is pseudocolored for better calcium visualization (blue = low, red = high).
(10.89 MB MOV)

Movie S9 IP_3_R-3 siRNA reveals its involvement in calcium store depletion upon exposure to CVB3. HBMEC monolayers were transfected with IP_3_R-3 siRNA, loaded with Fura-2AM, and exposed to CVB3 (MOI = 50) after 1 min. Movie was pseudocolored for better calcium visualization (blue = low, red = high).
(10.48 MB MOV)
