# Supplementary figures and images for: Release of Intracellular Calcium Stores Facilitates Coxsackievirus Entry into Polarized Endothelial Cells
Source: PLoS Pathog. 2010 Oct 7;6(10):e1001135. doi: 10.1371/journal.ppat.1001135 (PMC2951373; doi:10.1371/journal.ppat.1001135)

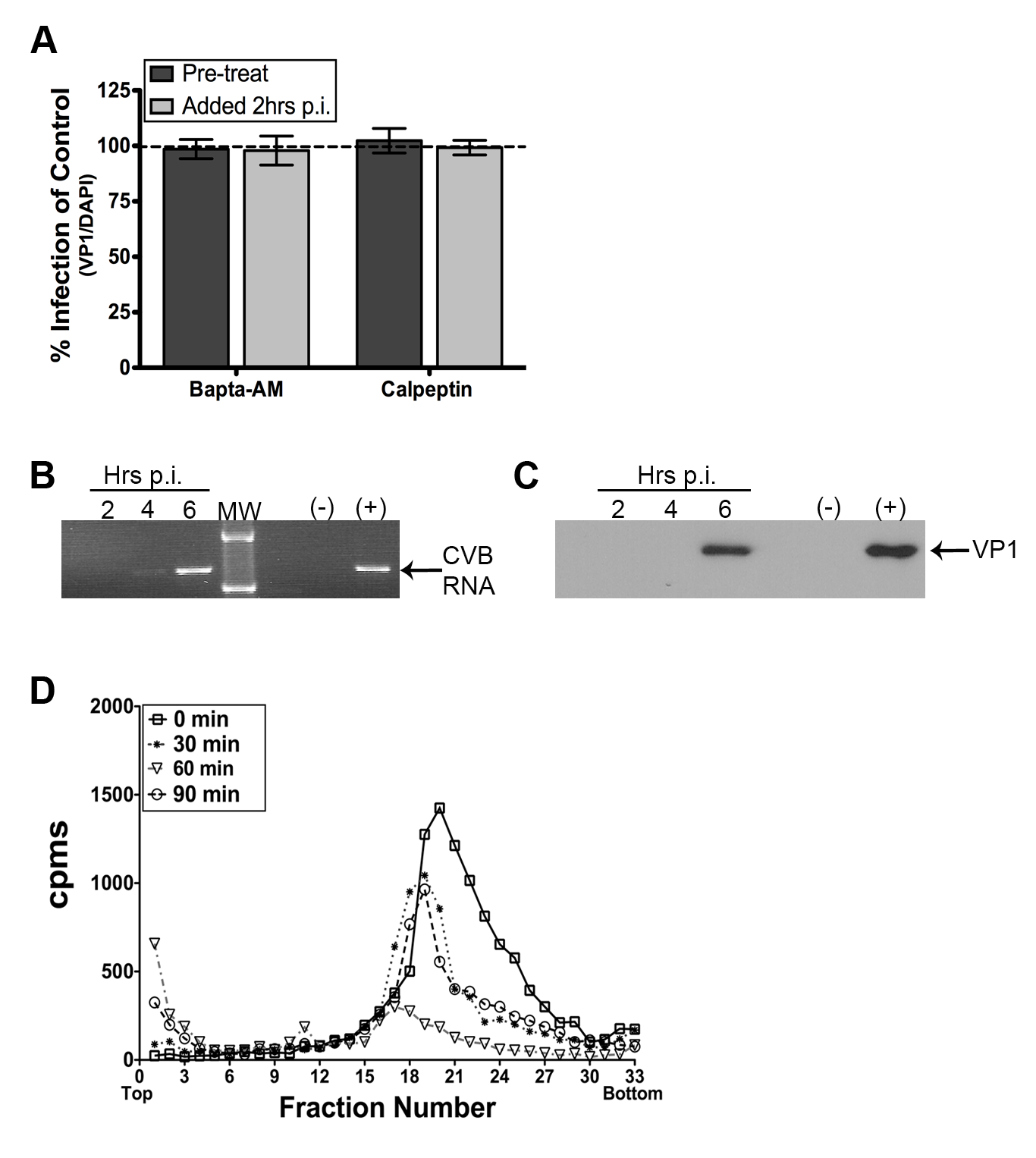

Supplement: Figure S1 — CVB-induced Cai 2+ depletion occurs prior to uncoating and replication. (A) HBMEC were treated with Bapta-AM and infected with VSV (MOI = 1) for 8 hrs. Inhibitor was added to cultures 1 hr before (pre-treat) or 2 hrs after (post-treat). The graph indicates the percentage of cells expressing VSV-G compared to control (dashed line). (B) RT-PCR or (C) Western blot analysis of RNA/protein collected from HBMEC infected with CVB (10 PFU/cell) for the indicated times. Negative [(-) no infection] and positive [(+) overnight infection with CVB] are shown. (D) 35S-labeled virus particle at various stages of internalization were recovered by cell lysis with sucrose gradient lysis buffer (10mM Tris-HCl, pH 7.6, 1mM NaCl, 1mM EDTA, 1% NP40, 0.5% sodium dodecyl sulfate (SDS). Cell lysates were overlaid on linear 15-30% sucrose gradients and centrifuged at 39,000 rpm for 150 min at 4°C in a Beckman SW41Ti rotor. Fractions (400 µl) were collected from the top of the gradient and radioactivity was measured. (0.32 MB TIF) [file ppat.1001135.s001.tif]

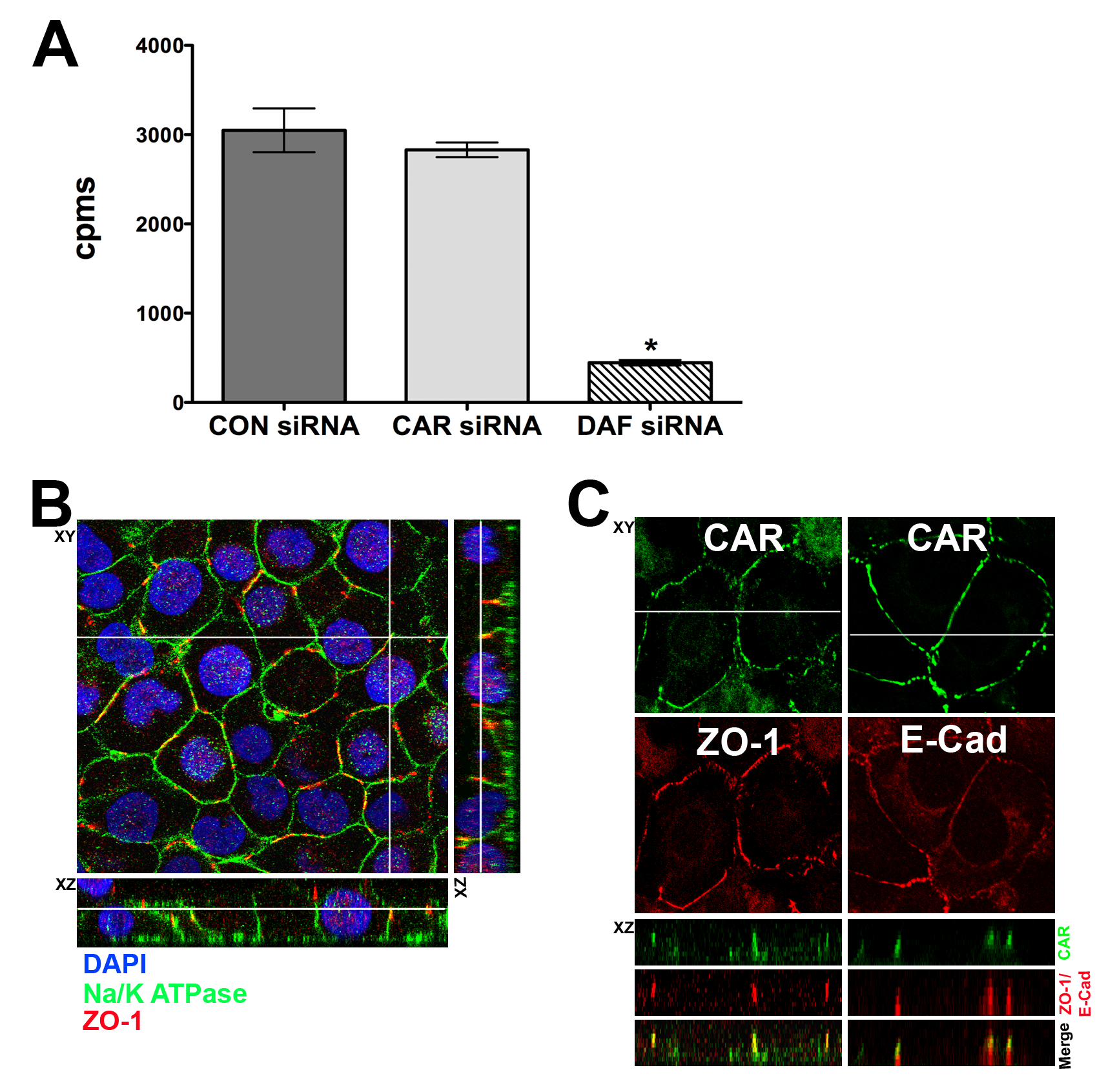

Supplement: Figure S2 — CAR is sequestered in the tight junctions of HBMEC. (A) HBMEC were transfected with control, CAR, or DAF siRNAs and exposed to S35-labeled CVB (12,000 cpms) at 16°C for one hour. Following binding, cells were washed, lysed, and radioactivity was counted. (B) Confocal micrographs of HBMEC immunostained for the basolateral-localized Na+/K+ ATPase pump (green) and the tight junction marker ZO-1 (red) (DAPI - blue). (C) Confocal micrographs of CAR (green) and ZO-1 (red, left), or E-cadherin (red, right). (1.97 MB TIF) [file ppat.1001135.s002.tif]

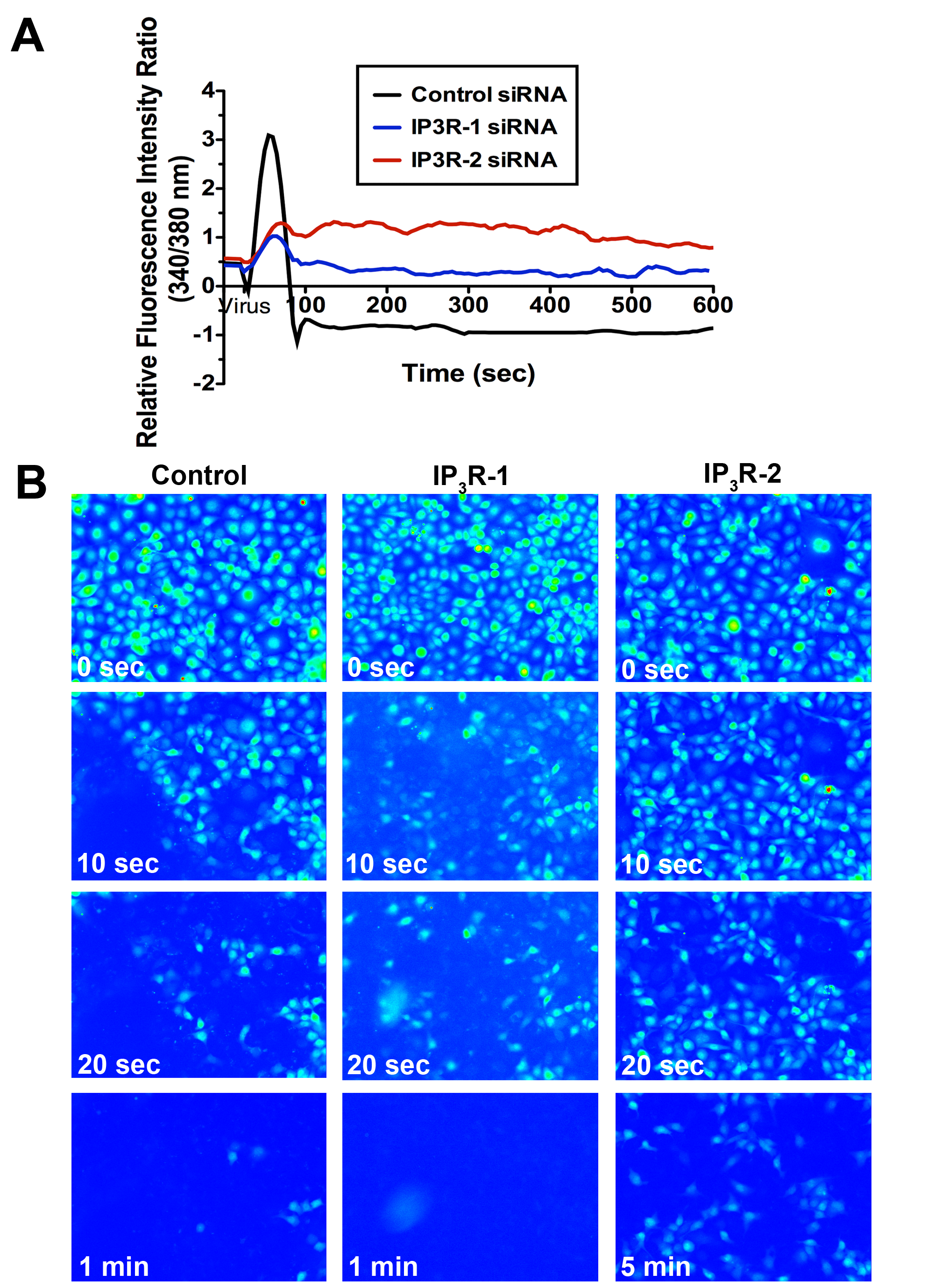

Supplement: Figure S3 — IP3R-1 and -2 siRNAs have modest effects on CVB-induced Cai 2+ mobilization. (A) Intensity ratio graph of HBMEC transfected with control, IP3R-1, or IP3R-2 siRNAs, loaded with Fura-2AM and exposed to CVB (55 sec). (B) Still images of Fura-2-loaded HBMEC transfected with control, IP3R-1, or IP3R-2 siRNAs and exposed to CVB. (3.27 MB TIF) [file ppat.1001135.s003.tif]

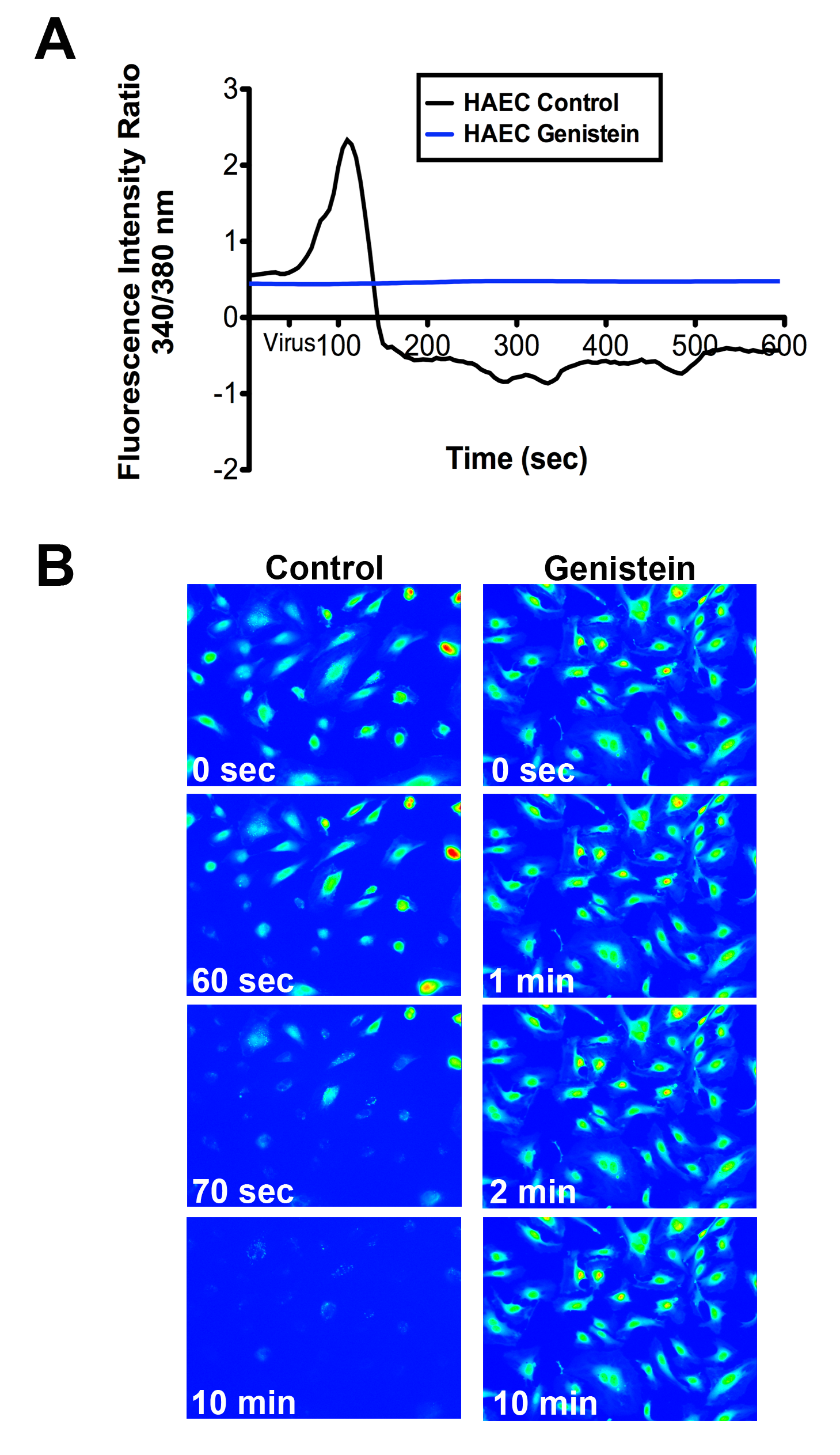

Supplement: Figure S4 — Tyrosine kinases are required for CVB-induced Cai 2+ release in HAEC. (A) Intensity ratio graph of HAEC pre-treated with control (no inhibitor) or genistein and exposed to CVB (55 sec). (B) Still images of Fura-2 loaded HAEC with or without genistein and exposed to CVB. (1.54 MB TIF) [file ppat.1001135.s004.tif]

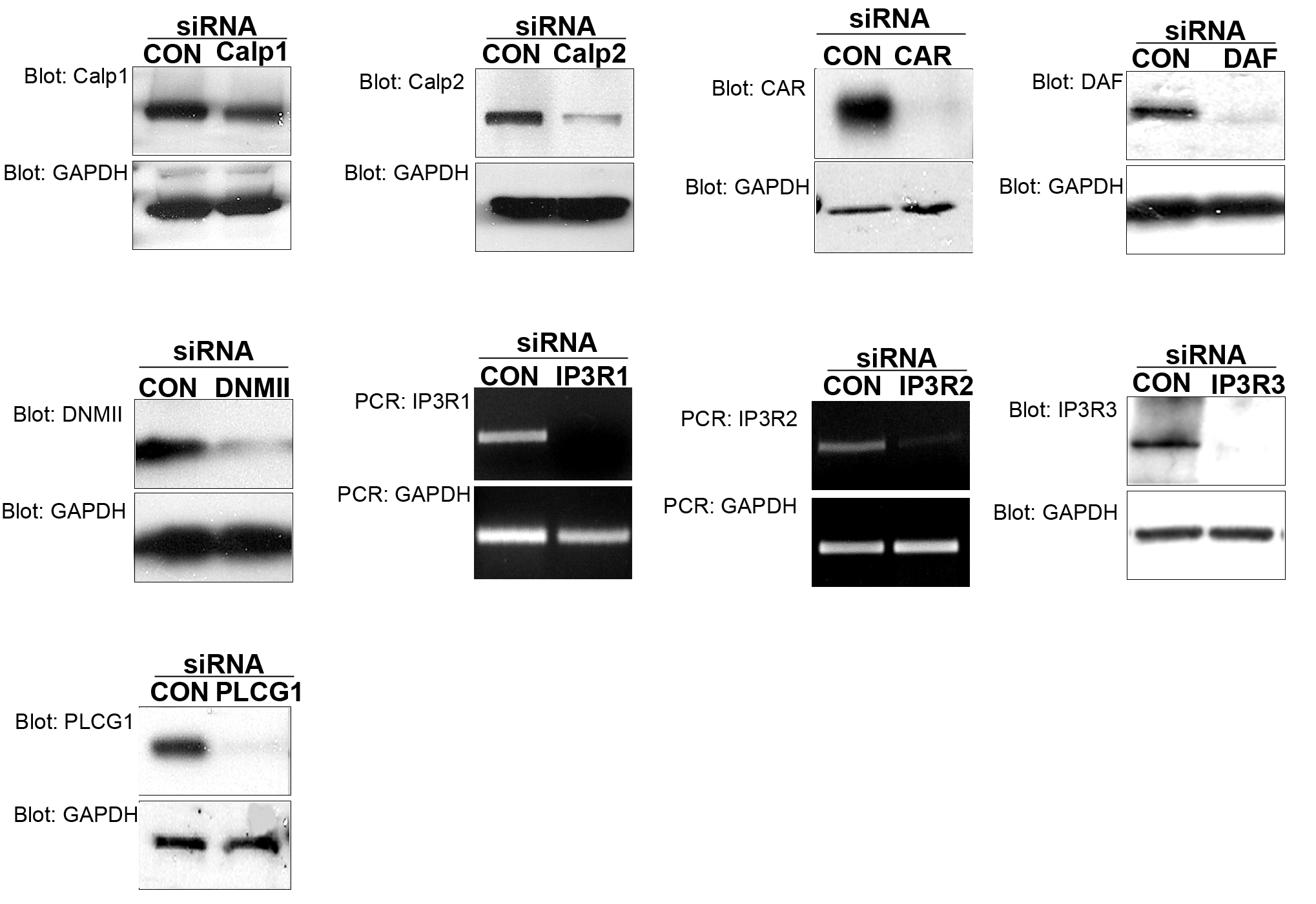

Supplement: Figure S5 — Efficacy of siRNA silencing in HBMEC. Western blot or RT-PCR analysis in HBMEC transfected with the indicated siRNAs: control (CON), calpain-1 (CALP1), calpain-2 (CALP2), CAR, DAF, dynamin II (DNMII), IP3R1, IP3R2, IP3R3, or PLCγ1 (PLCG1). For immunoblots, membranes were stripped and reprobed with GAPDH as a loading control. For RT-PCR, cDNA was amplified using GAPDH primers. (0.39 MB TIF) [file ppat.1001135.s005.tif]

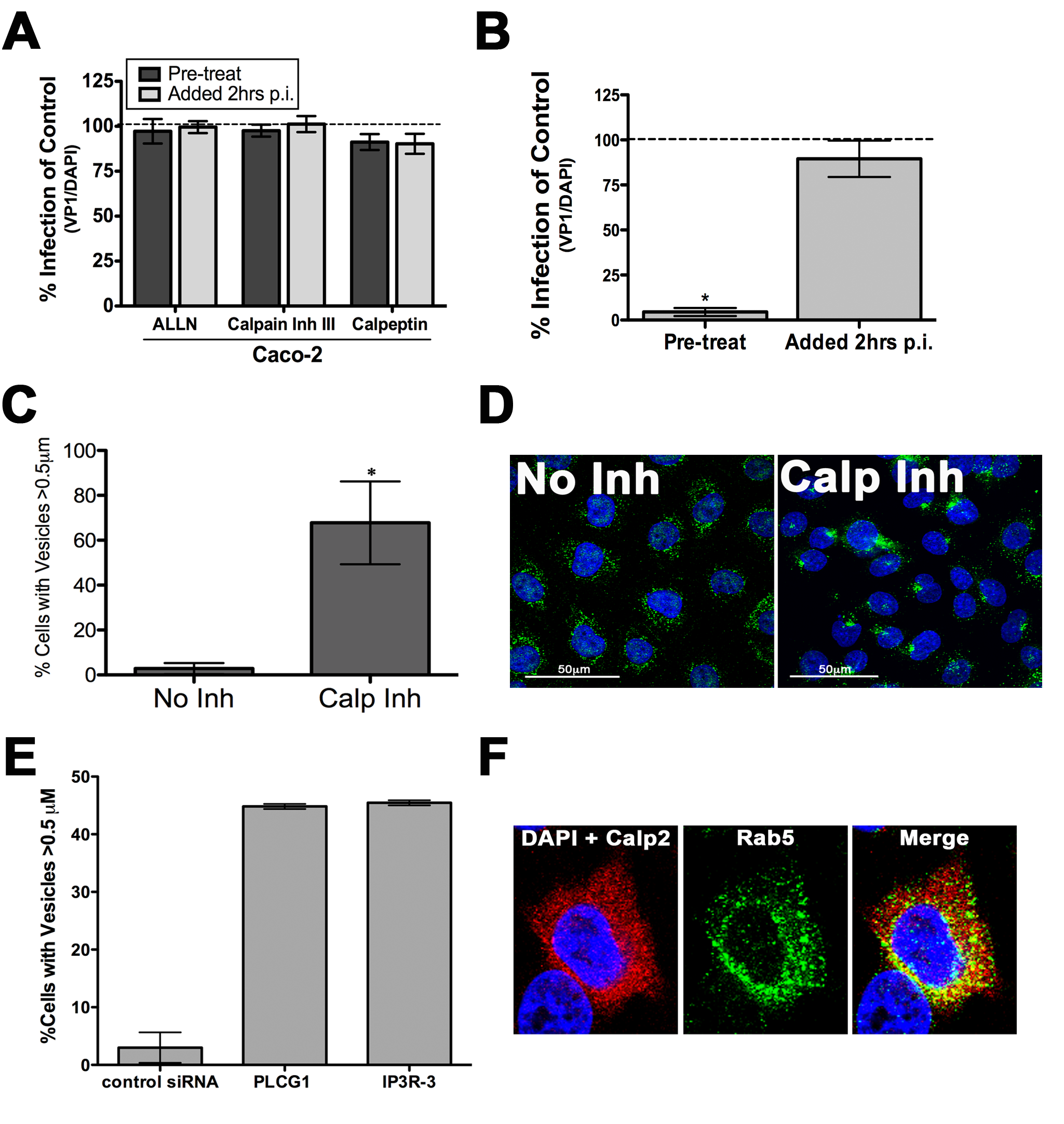

Supplement: Figure S6 — Calpain-2 is required for viral trafficking in HAEC and HBMEC. (A) Caco-2 monolayers were treated with the indicated calpain inhibitors and infected with CVB (MOI = 1) for 7hrs (Caco-2). Inhibitor was added to cultures 1 hr before infection (pre-treat) or 2 hrs p.i. Dashed line indicates the infection level of control cells. (B) Primary HAEC cells were treated with calpain inhibitor III and infected with CVB. Inhibitor was added to cultures 1 hr before infection (pre-treat) or 2 hrs p.i. (C) Quantification of vesicles (with diameter >500nM) in HBMEC in the absence or presence of calpain inhibitors. Data are presented as the percent of total cells containing vesicles >500nM in diameter (total number of cells counted - 75 for no inhibitor and 117 for calpain inhibitors). (D) Representative images of the quantification shown in (C). VP1 (green) and DAPI (blue). (E) Quantification of vesicles (with diameter >500nM) in HBMEC transfected with control siRNA, PLCG1 siRNA, and IP3R-3 siRNA. (F) Confocal images of HBMEC stained for calpain 2 (red) and stained with mouse monoclonal Rab5 GTPase (green) (2143, Cell Signaling Technology). (1.63 MB TIF) [file ppat.1001135.s006.tif]

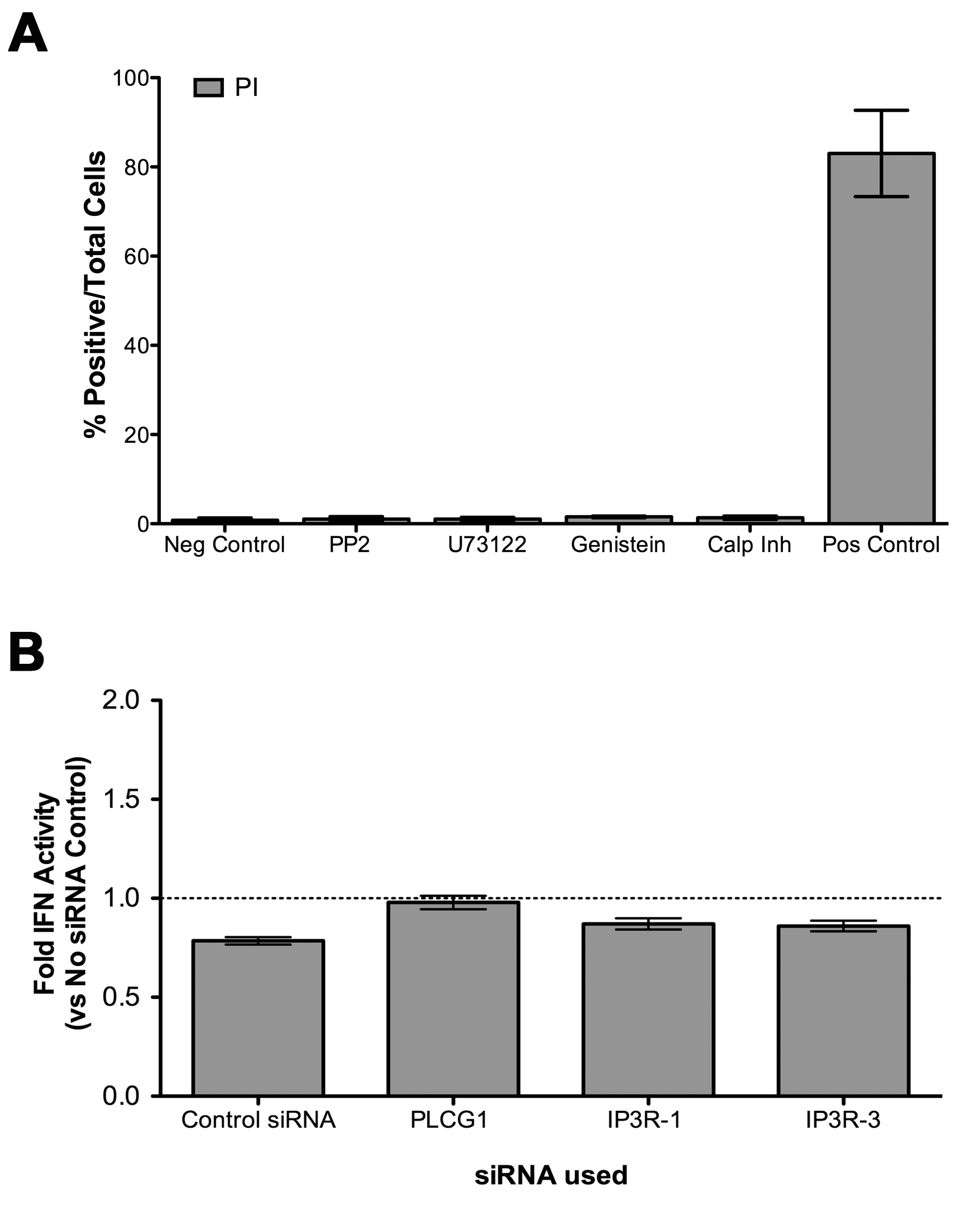

Supplement: Figure S7 — Toxicity panels for pharmacological inhibitors and siRNAs. (A) Extent of PI uptake in HBMEC following 7 hr incubation with the indicated inhibitors. Toxicity was calculated as the percent of cells positive for PI/total cells. (B) Induction of type I interferon signaling in HBMEC transfected with a luciferase reporter plasmid and then select siRNAs. Data are presented as a fold increase in comparison to control (no siRNA) levels. (0.93 MB TIF) [file ppat.1001135.s007.tif]
